# Complete Genome Sequence, Molecular Characterization and Phylogenetic Relationships of a Novel Tern Atadenovirus

**DOI:** 10.3390/microorganisms10010031

**Published:** 2021-12-24

**Authors:** Alina Matsvay, Marina Dyachkova, Ivan Mikhaylov, Daniil Kiselev, Anna Say, Valentina Burskaia, Ilya Artyushin, Kamil Khafizov, German Shipulin

**Affiliations:** 1 Federal State Budgetary Institution “Centre for Strategic Planning and Management of Biomedical Health Risks” of the Federal Medical Biological Agency, 119121 Moscow, Russia; MDyachkova@cspmz.ru (M.D.); IMkhaylov@cspmz.ru (I.M.); ASay@cspmz.ru (A.S.); shipgerman@gmail.com (G.S.); 2 Moscow Institute of Physics and Technology, National Research University, 115184 Moscow, Russia; kkhafizov@gmail.com; 3 Institute for Neurosciences of Montpellier, University of Montpellier, INSERM, 34091 Montpellier, France; daniil.kiselev@inserm.fr; 4 Skolkovo Institute of Science and Technology, 143026 Moscow, Russia; valya.burskaya@gmail.com; 5 Faculty of Biology, Lomonosov Moscow State University, 119991 Moscow, Russia; sometyx@gmail.com

**Keywords:** *Adenoviridae*, *Atadenovirus*, tern atadenovirus 1, genome annotation, phylogenetics

## Abstract

Discovery and study of viruses carried by migratory birds are tasks of high importance due to the host’s ability to spread infectious diseases over significant distances. With this paper, we present and characterize the first complete genome sequence of atadenovirus from a tern bird (common tern, *Sterna hirundo*) preliminarily named tern atadenovirus 1 (TeAdV-1). TeAdV-1 genome is a linear double-stranded DNA molecule, 31,334 base pairs which contain 30 methionine-initiated open reading frames with gene structure typical for *Atadenovirus* genus, and the shortest known inverted terminal repeats (ITRs) within the *Atadenovirus* genus consisted of 25 bases. The nucleotide composition of the genome is characterized by a low G + C content (33.86%), which is the most AT-rich genome of known avian adenoviruses within *Atadenovirus* genus. The nucleotide sequence of the TeAdV-1 genome shows high divergence compared to known representatives of the *Atadenovirus* genus with the highest similarity to the duck atadenovirus 1 (53.7%). Phylogenetic analysis of the protein sequences of core genes confirms the taxonomic affiliation of the new representative to the genus *Atadenovirus* with the degree of divergence from the known representatives exceeding the interspecies distance within the genus. Thereby we proposed a novel TeAdV-1 to be considered as a separate species.

## 1. Introduction

Adenoviruses (AdVs) are common pathogens capable of replicating in almost all classes of vertebrates [1]. The family is divided into 6 genera: *Atadenovirus, Aviadenovirus*, *Ichtadenovirus*, *Mastadenovirus*, *Siadenovirus*, and recently accepted *Testadenovirus* [2].

Family members are medium-sized, non-enveloped viruses whose genetic information is contained in a double-stranded DNA molecule of variable size from 26 to 48 kb, the ends of which harbor inverted terminal repeats (ITR) found in all AdVs [3]. The genomes of all AdVs have a similar structure. The central part of the genome consists of a conservative set of genes encoding household proteins that are necessary for the implementation of the life cycle of the virus. They are required for viral DNA replication (DNA polymerase-DNApol, terminal protein precursor-pTP, DNA-binding protein-DBP), involved in DNA encapsidation (52 K and IVa2 proteins), and building virion architecture (penton base, hexon, fiber, pIIIa, pVI, pVII, pVIII, pX, protease, 100 K protein, and 33 K protein) [4]. *Atadenovirus* representatives additionally have genus-specific genes for p32 K and LH3 (E1B homolog) proteins [5]. At the end of the DNA molecule, there is a variable region, whose sequence is usually unique for each species.

Birds are common hosts for AdVs of various genera, including *Aviadenovirus*, *Atadenovirus,* and *Siadenovirus*, many of which are pathogenic and often cause deadly diseases [1]. Infections carried by migratory birds can potentially spread over significant distances. It has been suggested that under some conditions, AdVs could be more virulent in non-host-adapted species than in their typical host [6]. A recent global systematic review carried out by Borkenhagen and colleagues [7] demonstrated substantial evidence suggesting AdVs have previously crossed host species barriers and this is likely to be repeated in the future. In some cases, the cross-species transmission of viruses may have large-scale consequences, such as, for example, global pandemics of fatal infectious diseases. A striking example is the relatively recent panzootic bird flu caused by the highly pathogenic avian influenza virus of type H5N1 [8]. In the human population, outbreaks of zoonotic viral infections have often escalated into extremely dangerous epidemics on a global scale, for example, the 2009 swine flu pandemic caused by the swine influenza virus of type H1N1 [9,10], as well as the ongoing COVID-19 pandemic caused by the SARS-CoV-2 coronavirus [11,12], and multiple others. Such pandemics are not only highly lethal but have serious socio-economic consequences. Therefore the discovery and study of infectious agents of potential superspreaders [13] are tasks of high importance.

During the present study, we sequenced, assembled, and characterized the first complete genome of a new, previously undescribed tern adenovirus—tern atadenovirus 1 (TAdV-1) isolated from a bird, common tern (*Sterna hirundo*) as well as determined its taxonomic position and phylogenetic relationships with other currently known AdVs. We also studied the molecular evolution of the core genes of AdVs in the genome of the TeAdV-1 and the genomes of *Atadenovirus* in general. Analysis of positive selection helped us to elucidate the evolutionary processes occurring on the background of divergence, speciation, and adaptation of the virus to the host. To date, tern adenoviruses have not yet been described in publications.

## 2. Materials and Methods

### 2.1. Sampling

The sample used in this study belongs to the Common tern (*Sterna hirundo)* and was part of the collection of biological samples obtained from migratory birds in the near-water complex [14]. Bird droppings were collected on the banks of the Yenisei River near the village of Mirnoye (Russia, Siberia, Krasnoyarsk Region). The collection of samples was carried out without direct contact with animals, no invasive interventions with animals were performed.

Samples of biological materials were placed into sterile tubes with a transport medium (Reagent for transportation and storage of clinical material, Amplisens, Moscow, Russia) and transported to the laboratory within 48 h where they were stored in a low-temperature refrigerator (minus 82 °C) until required for the experiment.

### 2.2. Sample Preparation and Sequencing

Nucleic acids were isolated with Allprep DNA/RNA mini kit (Qiagen, Hilden, Germany) following the manufacturer’s instructions and used for all subsequent procedures.

Preliminary screening for common viral pathogens was carried out by multiplex PCR with a pool of 26 primer pairs and subsequent sequencing of obtained amplicons on the Ion S5 System (Thermo Fisher Scientific, Waltham, MA, USA) as described earlier [14].

Extracted DNA was used for library preparation with NEBNext Ultra II DNA Library Prep Kit (New England Biolabs, Ipswich, MA, USA) in accordance with the manufacturer’s protocol after preliminary ultrasonic fragmentation using M220 Focused-ultrasonicator (Covaris, Woburn, MA, USA). Sequencing was performed on the Illumina MiSeq platform (Illumina, San Diego, CA, USA) with MiSeq Reagent Kit v2 (500-cycles) (Illumina, USA). A total of 1,462,033 paired-end reads were obtained for this sample.

### 2.3. Assembly and Genome Annotation

The de novo assembly was performed using the SPAdes software v.3.15.3 [15] with the “careful” option activated. A total of 24,465 contigs were obtained, ranging in length from 128 to 14,540 nucleotides, of which 32, with length >1000 nucleotides and mean coverage >100, were chosen for further analysis. Fifteen of them were attributed to the representative genome of the *Adenoviridae* family by taxonomic classification of nucleotide and translated protein sequences. Taxonomic classification of nucleotide and translated protein sequences was carried out using the BLAST algorithm [16] and the NCBI Taxonomy database [17]. These contigs (maximum length 14,540 nucleotides) were used to obtain whole-genome assembly using the SeqMan NGen program (DNASTAR, Madison, WI, USA).

To evaluate the assembly quality and correct possible errors, the original reads were mapped to the resulting assembly. Mapping was carried out using the BWA v.0.7.17 [18]. Samtools package v.1.10 [19] was used for operations with sam/bam files. The assembly was checked for single nucleotide errors, short insertions, deletions, and breakpoints (stacks of soft-, hard-clipped reads) using Tablet program v.1.19.09.03 [20]. Separately, the quality and integrity of the 3′ end of the assembly were assessed.

We then used MUMmer v.3.0 [21] to verify the resulting nucleotide sequence for possible assembly artifacts. A Viral Genome Annotation System Vgas [22] was used to annotate the assembly as well as to reannotate genomes retrieved from public databases (listed below). Orthofinder v.2.5.4. was used to determine the core genome of the studied set of AdVs of various species [23]. Genes are known to have splicing were manually re-annotated as follows: (1), all possible splice sites in the TeAdV-1 genome were predicted using the BDGP Splice Site Prediction by Neural Network [24] (Supplementary Table S1); (2) in the region where the beginning of the first exon of the corresponding gene was expected (based on the analysis of genome structure of adenoviruses), all possible start codons were identified; (3) using the BioPyton library [25], all possible protein sequences were built from each start to each end of the exon of the predicted splice donor site, then-from the beginning of the second exon of each possible acceptor splice site to the first occurrence of the stop codon; (4) all obtained candidate-sequences were checked using BLAST search [16] and the candidate with the best “%cover” and “identity” scores to the homologous proteins was selected.

### 2.4. Phylogenetic Analysis

The genome assemblies used in the analyses were retrieved from GenBank [26] (date of accession 17 August 2021). To build a phylogenetic tree of the representatives of the *Adenoviridae* family, we accessed the International Committee on Taxonomy of Viruses database (ICTV, date of accession 17 August 2021), which lists viruses that can serve as representative members of each species [2]. Sixty-three genome assemblies of different species of AdVs, chosen with the assistance of ICTV (Supplementary Table S2), were used. To build a phylogenetic tree of the representatives of the *Atadenovirus* genus, we used nucleotide assemblies of 25 *Atadenovirus* representatives with the complete genome sequence.

The multiple interspecies alignments of the 63 amino acids sequences of the 12 core proteins were performed independently in the ClustalW program [27] implemented in the MEGA-X software v.10.2.4 [28] using default settings. To eliminate poorly aligned and diverged regions, Gblocks v.0.91b [29] was used with the default parameters and the resulting fragments were concatenated. Before phylogenetic analysis, the best-fit partitioning scheme and the substitution models for each partition were determined using PartitionFinder v.2.1.1 [30] under the corrected Akaike (AICc) and the Bayesian (BIC) information criteria.

The maximum likelihood unrooted tree was generated using RAxML-NG v.1.0.2 [31]. Bootstrapping converged after 100 replicates. The obtained phylogenetic tree was rooted using the midpoint rooting method implemented in FigTree v.1.4.4 [32].

The nucleotide sequences of the genomes of 25 representatives of the genus *Atadenovirus* (Supplementary Table S3) were used to construct multiple interspecies alignments of 12 core genes. We used the nucleotide sequences of the core genes to construct the phylogeny of *Atadenovirus*, since we discovered resolution problems at the genus and/or species level while using protein sequences. The multiple codon alignments were performed independently as described above. Further analysis was carried out in a similar way, except for the choice of an evolutionary model. In particular, the most parameter-rich model GTR + I + G [33,34] with 12 partitions was used. The complete genome sequence of red squirrel adenovirus 1 (*Squirrel mastadenovirus A*, GenBank accession is KY427939.1) was used for an outgroup rooting. The trees were visualized using iTOL v.6 [35].

### 2.5. Comparative Analysis

For comparative analysis, we created pairwise alignments of (1) whole-genome sequences and (2) amino acid sequences of core genes of representative genomes of each *Atadenovirus* species (Supplementary Table S4) and TeAdV-1 using MAFFT [36] for every possible pair of genomes and proteins. Pairwise identity for each alignment was calculated using the AlignIO module of BioPython using DistanceCalculator from Bio.Phylo.TreeConstruction module of BioPython [25] using ‘Identity’ model for calculation.

### 2.6. Species Delimitation

We used three approaches that propose *de novo* species partitions to confirm the species status of the virus under the study. First, we used the GMYC method [37]. The GMYC is one of the most popular coalescent-based species delimitation methods, designed for single-locus data [38,39,40] and previously used to describe new species [41]. The method separately models the fit of Yule (pure birth; [42]) and coalescent processes [43] to an ultrametric tree to define the transition from species-level to population-level processes, used to delimit evolutionarily significant units. We used an ultrametric tree as input. The ultrametric timetree was obtained by applying the RelTime method [44,45] implemented in the MEGA-X software v.10.2.4 [28] using the GTR + I + G model [46].

We also implemented the bPTP method [37], using the maximum-likelihood phylogeny as the input tree. The bPTP method is an updated version of the original PTP with Bayesian support values. The PTP is a method that models speciation and coalescent events relative to numbers of substitutions rather than time, and uses heuristic algorithms to identify the most likely classification of branches into the population and species-level processes, used to delimit ESUs. This approach assumes either substitutions are clocklike or, if substitution rates vary across the tree, that coalescent and speciation events occur at a constant rate per substitution event, rather than per unit of time. The key advantage of the PTP, however, is that it is devised for non-ultrametric trees [37]. We ran the bPTP analysis for 500,000 steps, with default parameters.

Finally, we used ASAP [47] in default mode for species delimitation. The ASAP algorithm is an ascending hierarchical clustering, merging sequences into “groups” based on pairwise genetic distances, that are successively further merged until all sequences form a single group. A matrix of patristic distances obtained using the T-Rex web server [48] was used as input. The partition with the best asap score was selected.

Additionally, we used Birky 4x index [49] to validate the species delimitation results of the two *Psittacine atadenovirus A* strains. The method is based on the theory that random genetic drift in single species leads to the formation of clades and singlets, which descended from a common ancestor on average 2Ne generations ago (Ne is the effective population size), and their distance from each other will be less than 2Ne generations. Alternatively, as a result of the speciation process, a species is divided into completely separated populations, which form clusters separated by a gap exceeding 2Ne. According to the 4 × rule, clusters separated by t ≥ 4Ne generations are characterized by a probability of less than 5% that the clusters were formed as a result of random genetic drift. Unfortunately, the Birky index cannot be applied to species delimitation of clades, at least one of which contains only one sequence, because it becomes impossible to calculate intra-clade distances.

### 2.7. Codon-Based Analysis of Positive Selection

The analysis of possible recombination events was performed using the GARD program [50] implemented in the HyPhy software v.2.5 [51].

To examine the impact of pervasive positive selection on the set of adenoviral core genes in the genomes of *Atadenovirus* representatives, we used both the CODEML program as implemented in PAML software package v.4.9 [52] and the FEL method [53] as implemented in HyPhy software package v.2.5 [51]. Site models (M8 and M8a) were executed in CODEML, and then statistical likelihood ratio tests (LRT) were performed to evaluate adaptive evolution acting at particular sites along all lineages of the phylogenetic tree. When the LRT was significant, the codons that were likely to evolve under positive selection based on PP thresholds of 0.7 and 0.95 were filtered out. Further, to obtain reliable analysis results, we found an intersection between statistically significant sites acquired using both methods. The conversion of the codon coordinates of the alignment, consisting of concatenated blocks, back to the original coordinates of the amino acid sites of proteins was performed using a custom Python script.

To examine the impact of episodic positive selection on the set of adenoviral core genes in the TeAdV-1 genome, we used both the CODEML program as implemented in the PAML software package v.4.9 [52] and BS-REL approach as implemented in HyPhy software package v.2.5 [51]. We used a branch-site likelihood method known as test 2 which is recommended by the authors as a direct test for positive selection at the molecular level [54]. Branch-site models (null and alternative, which are A1 and A respectively in CODEML) were executed and then statistical likelihood ratio tests (LRT) for evaluating adaptive evolution in the linage of interest were conducted. Filtration and the intersection of statistically significant sites occurred according to the pipeline described above.

### 2.8. Protein 3D Structure Prediction

To model 3D structures of TeAdV-1 proteins we used AlphaFold2 [55] with Uniref90, Mgnify, BFD, UniClust30, and pdb70 databases. The prediction for each protein comprised of 5 relaxed models, 5 unrelaxed models, and 5 resulting models. All models were compared with each other using the “match maker” function of UCSF Chimera [56] to evaluate the uniformity of predictions. We used the capsid structure of lizard adenovirus 2 [57] and human adenovirus 41 [58] as templates for capsid structures modeling. Visualization for all individual proteins and structures was performed using UCSF Chimera [56].

## 3. Results

### 3.1. Genome of TeAdV-1 and Comparative Analyses

**Figure 1:**
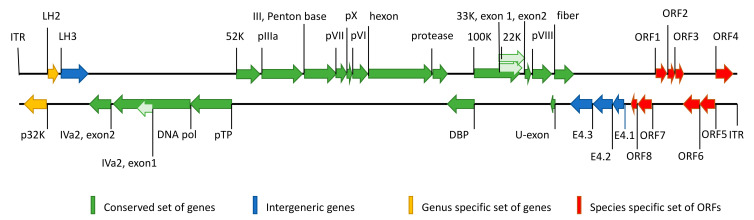
Schematic view of the TeAdv-1 genome structure. Genes and ORFs are illustrated as arrows pointing in the direction of transcription. Green indicates a conserved set of genes present in the genomes of AdVs. Genes that are found in the genomes of more than one genus of AdVs, including the genus *Atadenovirus*, are shown in blue. Genes specific to *Atadenovirus* are highlighted in yellow. Specific for TeAdV-1 genes colored red.

Tern atadenovirus 1 (TeAdV-1) genome is a linear double-stranded DNA molecule, 31,334 base pairs in length. The genome shows typical for AdVs structure and has 30 methionine-initiated open reading frames (ORFs), 22 of which were identified as protein-coding genes by comparative analysis of homologs protein sequences. Similar to other AdVs, TeAdV-1 contains a conservative set of genes located at the central part of the genome and species-specific end (Figure 1, Table 1). We identified the entire set of genes conserved for the *Adenoviridae* family, as well as genus-specific genes typical for *Atadenovirus* and 8 additional ORFs, the set of which is unique for TeAdV-1. Genes known to have splicing were manually annotated using predicted splice sites (Supplementary Table S2).

The coding region is surrounded by inverted terminal repeats (ITR), consisting of 25 bases with the coordinates of 1–25 sense orientation and 31,310–31,334 antisense orientation. To date, it is the shortest known ITR region within the *Atadenovirus* genus to the best of our knowledge and one of the shortest within all *Adenoviridae* family [60], closest by length to the *Siadenovirus* representatives (Supplementary Table S5).

**Figure 2:**
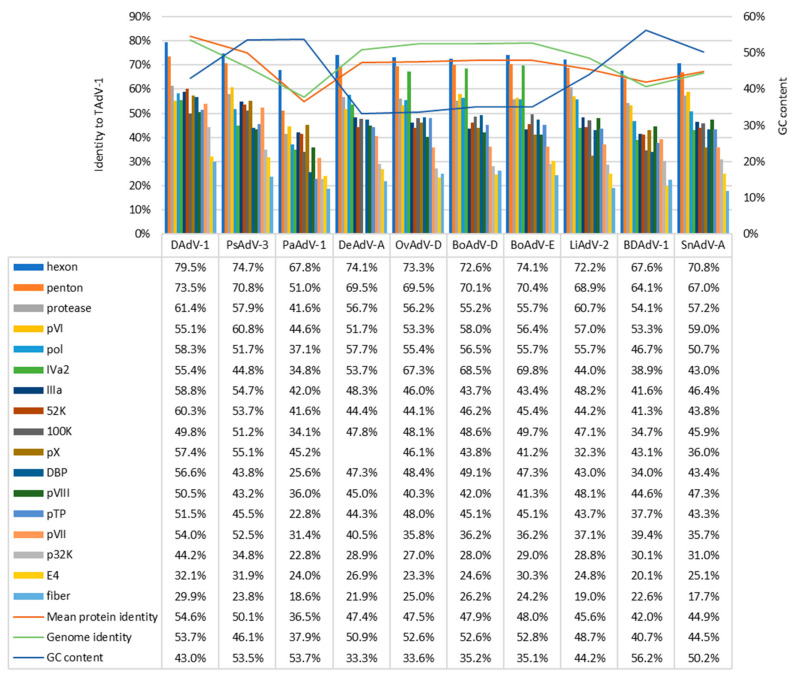
Comparison of TeAdV-1 genome with genomes of other members of *Atadenovirus* genus. Colored bars represent the degree of pairwise identity of specific proteins conservative within *Adenoviridae* family and *Atadenovirus* genus, colored lines represent mean protein and genomic sequence identity across all genes as well as mean GC content.

The nucleotide composition of the genome is characterized by a low G + C content-33.86%, which is more inherent in ruminant viruses than avian ones (Figure 2). However, the nucleotide sequence of the TeAdV-1 genome shows the closest resemblance to the duck atadenovirus 1 (KJ452172.1, *Duck atadenovirus A*) with identity 53.7% (Figure 2).

In general, the primary structure of proteins encoded by genes of the conserved region of the genome shows the maximum identity with DAdV-1, with the exception of the IVa2 protein, which is significantly more similar to ruminant viruses of the *Atadenovirus* genus (with an identity score being 10% higher), the pVI protein sequence of which appears to be closer to psittacine atadenovirus 3 (PsAdV-3, identity 5% higher), and 100 K protein which is also slightly more similar to PsAdV-3 (about 1% higher). *Atadenovirus* specific p32 K protein exhibited relatively low similarity ranging between 44 and 23% when calculated by an amino acid sequence where the highest identity score was also with DAdV-1. The most divergent protein was fiber with the highest identity to DAdV-1 (~30%) wheres conservative capsid proteins—penton base and hexon demonstrated the highest identity scores among all protein sequences: 79.5% and 73.5% to DAdV-1 respectively (Figure 2).

In the TeAdV-1 genome, we found three E4 genes (E4.1, E4.2, and E4.3). E4.2 and E4.3 proteins showed the highest similarity to the DAdV-1 proteins (pairwise identity 35.3 and 30.7% accordingly), whereas E4.1 protein was most similar with PsAdV-1 gene E4.1 with 29.1% amino acid sequence identity.

The variable region of the TeAdV-1 genome contains eight ORFs, 7 of which have sequence homology to ones found in other characterized *Atadenovirus* representatives. We have found homologs to hypothetical protein sequences for ORF1, 2, 3, and 7 in DAdV-1 and for ORF4, 5, 6 in PsAdV-3 with identity varies between 28 and 44% for different ORFs (Supplementary Table S6). ORF8 was not found among predicted protein sequences of other *Atadenovirus* species by blast search. ORF8 encodes short protein 80 amino acids in length, which appears to be unique for TAdV1 and consists of two alpha-helixes, connected with turn motif (HTH-motif).

### 3.2. Evolutionary Relationships of TAdV-1

In order to establish the relationship between TeAdV-1 and other members of the *Adenoviridae* family, we carried out a phylogenetic analysis. We used aligned concatenated sequences of core genes to build a phylogenetic tree. The use of sequences of concatenated genes as opposed to the use of sequences of individual genes increased the statistical power of the molecular evolution analysis and improved the accuracy of the obtained phylogenetic tree since a higher number of substitutions is analyzed. We constructed an *Adenoviridae* phylogenetic tree based on the concatenated amino acid sequences of the 12 core proteins that were found to be encoded in the genomes of all analyzed representatives of the family. The list of the core genes used for phylogenetic reconstruction and their annotations are presented in Table 2. Fiber protein was excluded from the set of core genes due to the low consistency of multiple sequence alignment arising from a high level of divergence of this protein.

**Figure 3:**
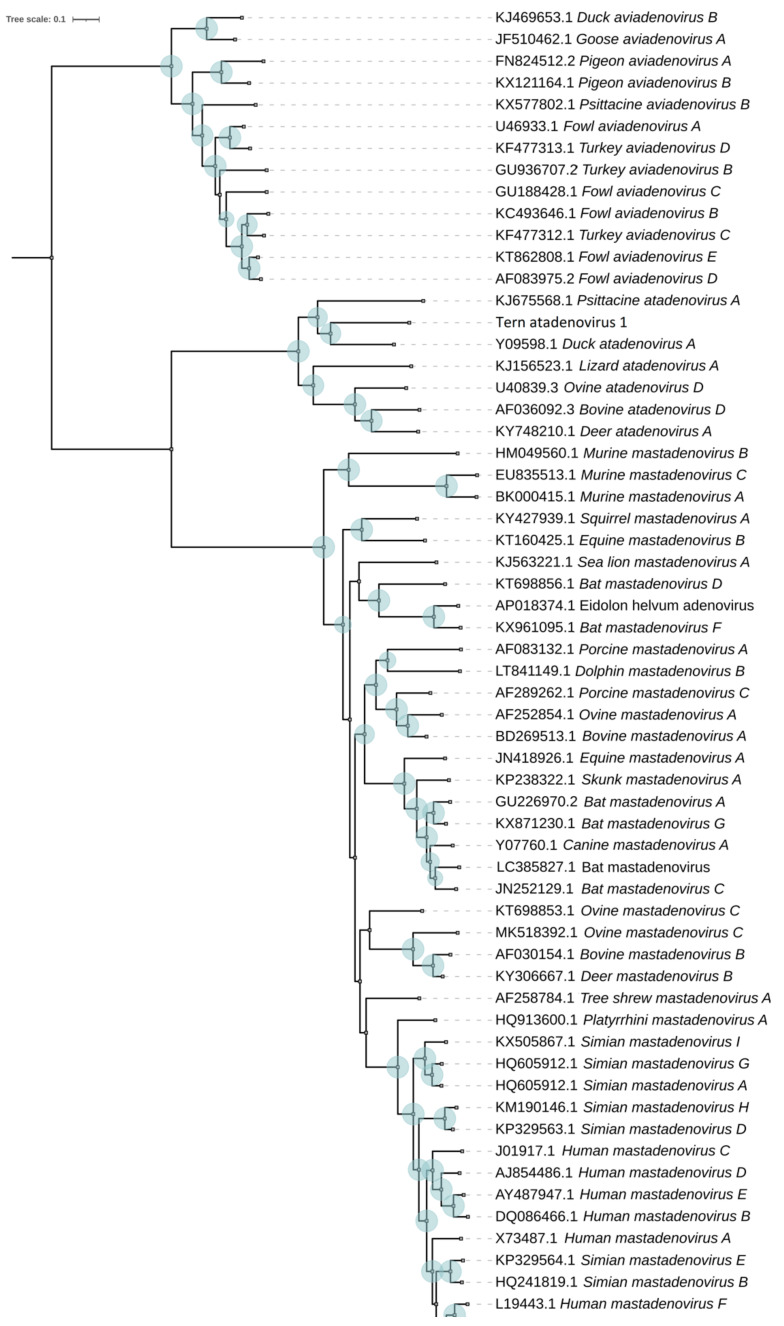
Maximum likelihood phylogenetic tree based on concatenated amino acid sequences of the 12 core proteins of 63 representatives of various species of the *Adenoviridae* family after 100 bootstrap replicates. Bootstrap values higher than 70 are marked next to the respective nodes (blue circles) showing a robust phylogenetic reconstruction.

The external nodes of the obtained phylogenetic tree were strongly supported by bootstrap values and, regardless of the slight differences, accurately reproduced the existing robust phylogenies of adenoviruses (Figure 3).

**Figure 4:**
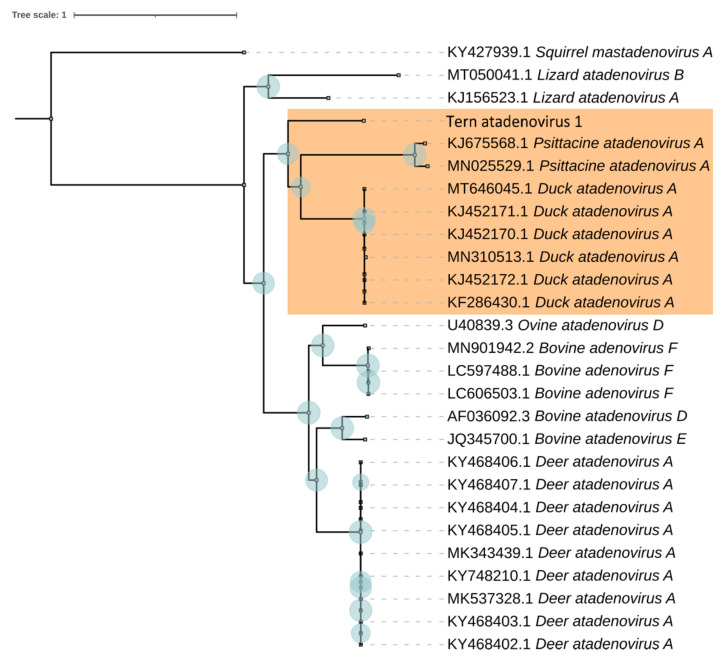
Maximum likelihood phylogenetic tree based on concatenated amino acid sequences of the 12 core proteins of 29 representatives of various species of the *Atadenovirus* genus after 1000 bootstrap replicates. Bootstrap values higher than 70 are marked next to the respective nodes (blue circles) showing a robust phylogenetic reconstruction. A clade of related species of avian viruses of the *Atadenovirus* genus is indicated (orange box).

Genome TeAdV-1 is located within the clade of the phylogenetic tree corresponding to the *Atadenovirus* genus. Based on the nucleotide sequences of the core genes, we constructed a phylogenetic tree of the *Atadenovirus* genus with a higher resolution (Figure 4).

For the species delimitation, we used several approaches. We used Assemble Species by Automatic Partitioning (ASAP) method to build species partitions from pairwise patristic genetic distances. As a result of using this method, the studied set of 27 *Atadenovirus* representatives was divided into 9 partitions corresponding to different species (Table 3). The obtained result demonstrates that the TeAdV-1 genome does not enter the partition together with other genomes. In addition, we used the bPTP web interface that delimits species based on the Phylogenetic Species Concept. We obtained the same result based on both the maximum likelihood and Bayesian approaches. Using both approaches, 11 species partitions were identified. In both cases, the TeAdV-1 forms an independent operational taxonomic unit. Finally, we applied the GMYC method. Twelve species groups were identified using a single-threshold approach and 8 species using a multiple-threshold approach (Table 3).

Thus, we have shown that the genome we are studying belongs to a new type of atadenovirus, and is not a strain of a previously sequenced or described species.

It is noteworthy that different methods delimit two *Psittacine atadenovirus A* strains differently. Therefore, we used an additional method (Birky 4 × rule) that also confirmed that the strains KJ675568.1 [59] and MN025529.1 [66] are different species.

### 3.3. Protein Prediction

**Figure 5:**
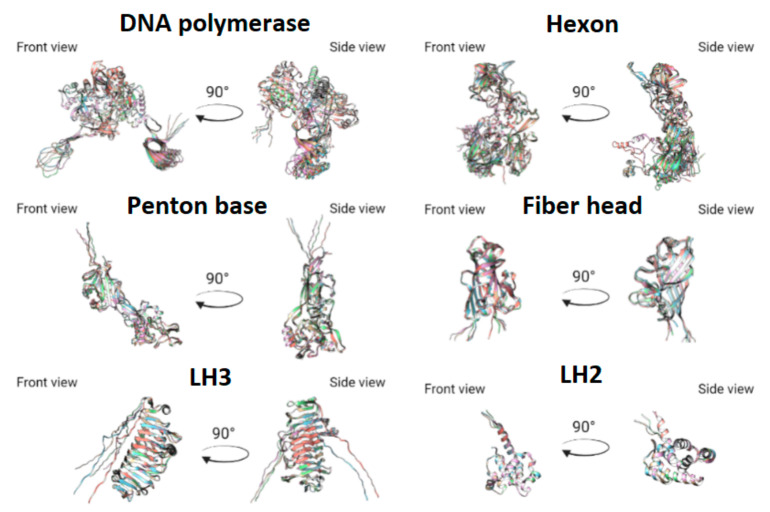
Comparison for predicted proteins 3D structures. For convenience, different colors were used for each predicted structure.

**Figure 6:**
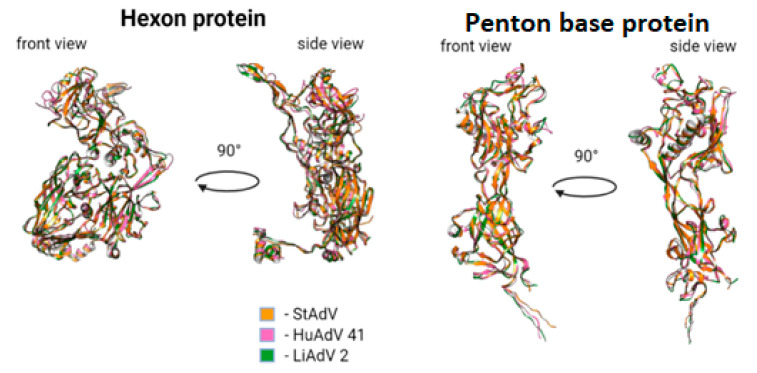
Predicted 3D structure of hexon and penton base proteins, compared to hexon and penton base proteins of HAdV-41 and LAdV-2.

We predicted 3D structures for DNA-polymerase, penton base, hexon, fiber, E1B large subunit, and E1B small subunit, using AlphaFold2. To evaluate uniformity between models predicted by AlphaFold2 we performed structural comparison using MatchMaker of UCSF Chimera. All domains of predicted proteins were highly similar in all models, apart from side chains, whose structures could not be predicted without information on protein-protein interactions (Figure 5 and Figure 6). Considering all of the above for fiber model comparison could be performed only for the head domain, as full protein could not be modeled with sufficient uniformity and precision as a monomer.

We also compared hexon and penton base with corresponding proteins of lizard adenovirus 2 (LAdV-2) [57] and human adenovirus 41 (HAdV-41) [58]. All motifs were conserved between all species with minor differences. For penton base protein we demonstrated the following differences:

1. Absence of alpha-helix, corresponding to Tyr288-Val285 in HAdV-41, lacking in TAdV, lacking in LAdV-2;
2. Elongation of alpha-helix Val138-Asn159 (21 aa) in TAdV, which corresponds to Glu173-Ala183 (10 aa) in HAdV-41 and Val138-Gly157 (19 aa) in LAdV-2;
3. Presence of beta-sheet-like short structure at Gly208-Asp210 in TAdV, lacking in HAdV-41 and LAdV-2 alike.
4. TAdV alpha-helix Thr41-Ser46, presented in HAdV-41 as Asn72-Ala75, lacking in LAdV-2.
5. Presence of structure Val233-Leu235 beta-sheet to Tyr236-Ile239 alpha helix, presented in StAdV which is absent in HAdV-41 and LAdV-2.

For hexon we demonstrated the following differences:

1. Presence of two beta-sheets Glu^380^-Gly^382^, Ala^400^-Ile^402^, absent in HAdV-41 and LAdV-2;
2. Elongation of beta-sheet Gln^816^-Cys^824^ (8 aa), corresponding to Val^816^ –Val^823^ (7 aa) in LAdV-2 and Ser^831^-Lys^836^ (5 aa)
3. Presence of beta-sheet Gln^229^-Leu^233^, absent in LAdV-2 and HAdV-41.
4. Elongation of beta-sheet Ser^187^-Ile^197^ (10 aa), corresponding to Arg^201^-Ile^203^ (2 aa) in HAdV-41 and absent in LAdV-2
5. Presence of alpha-helix Val^153^-Lys^157^, absent in HAdV-41 and LAdV-2.
6. Elongation of beta-sheet Cys^269^-Gly^273^ (4 aa), corresponding to Arg^263^-Thr^265^ (2 aa) in LAdV-2 and absent in HAdV-41.

**Figure 7:**
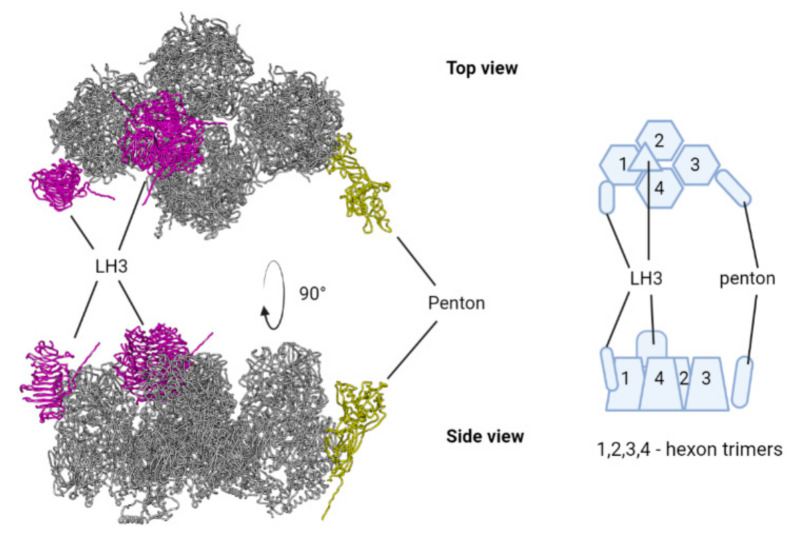
Icosaedral asymmetric unit (AU) of TAdV, penton base, and LH3 proteins are highlighted with color.

We used the capsid structure of lizard adenovirus 2 [57] and human adenovirus 41 [58] as templates for capsid structures modeling. We used UCSF Chimera MatchMaker to predict the 3D structure of TAdV capsid monomer, which consists of 4 hexon trimers in a diamond shape, penton base protein, 1 LH3 trimer, 1 LH3 monomer, pIIIa, pVIII, pVI, and pVII proteins, which is commonly referred to as icosahedral asymmetric unit (AU) (Figure 7) [57].

### 3.4. Detection of Adaptive Evolution Events

Since recombination is known to produce false-positive results [67], we screened the sequences for recombination events before running the positive selection tests. We found no evidence of recombination in the sequences studied. Then the analysis of molecular evolution was carried out using the method of maximum likelihood that allows for the detecting evolutionary events of pervasive or episodic positive selection in the nucleotide sequences of protein-coding genes.

#### 3.4.1. Pervasive Positive Selection in the Molecular Evolution of *Atadenovirus*

First, we tested the hypothesis for the presence of pervasive positive selection events in the molecular evolution of the adenoviral core genes. We obtained values of the log-likelihood function for the site models M8 and M8a using the CODEML program and then we conducted LRT for the presence of sites under positive selection pressure (ω > 1) in all branches of the phylogenetic tree of *Atadenovirus*. The LRT value for the test was statistically significant (LRT = 67.265, *p* << 0.01). Thus, in silico analysis showed that there is evidence for sites under the pressure of positive selection in all branches of the phylogenetic tree of *Atadenovirus* built on the basis of concatenated sequences of the adenoviral core genes. Then the specific sites were identified using the Bayes empirical Bayes (BEB) approach [43]. Sites with a posterior probability (*PP*) ≥ 0.7 were inferred to have evolved under positive selection. We found 7 positive selected sites with *PP* ≥ 0.7 in the genes encoding Pol, pTP, and III (penton base) (Supplementary Table S7). To test the robustness of our results, we used a complementary approach. We found pervasive positive selection events using the FEL method, which is consistent with our previous results. We found 10 sites under positive diversifying selection at *p* ≤ 0.1 in the genes encoding Pol, DBP, pIIIa (Supplementary Table S8).

#### 3.4.2. Episodic Positive Selection in the Molecular Evolution of TAtV-1

Episodic selection affecting individual sites in individual branches and clades of a phylogenetic tree is the most common case of positive selection. We tested the hypothesis that there are sites under the pressure of positive selection (ω > 1) in the tested branch compared to the other branches of the phylogeny. We obtained values of the log-likelihood function for two branch-site models A1 and A for the TeAdV-1 branch of the phylogenetic tree of *Atadenovirus* using the CODEML program, and then we applied LRT test 2 which was developed by the authors as a direct testing method for the detection of positive selection in the lineages of interest [40]. The LRT value for the test was statistically significant (LRT = 73.857, *p <<* 0.01). Thus, in silico analysis proved the presence of episodic positive selection events in the molecular evolution of the adenoviral core genes in the TeAdV-1 branch of the phylogenetic tree of *Atadenovirus*. We found 153 positive selected sites with *PP* ≥ 0.7. Again, the additional program was used to verify the obtained results. BS-REL models (null and alternative) were executed using HyPhy software. The LRT value for the test 2 was statistically significant (LRT = 12.907, *p* << 0.01). We detected 151 positive selected sites with *PP* ≥ 0.7. Eighty-six sites matched those previously predicted using CODEML (Supplementary Table S9). Sites with *PP* values ≥ 0.95 were inferred to be the most reliable candidates for positive selection (Table 4).

Candidate sites for positive selection, marked on the predicted 3D structures of the corresponding proteins, can be found in the supplementary material (Supplementary Figure S1).

## 4. Discussion

With this paper, we describe the first complete genome of tern adenovirus TeAdV-1, obtained from a bird *Sterna hirundo.*

The genome structure of TeAdV-1 corresponds to the general scheme of the genomes of AdVs: dsDNA molecule which contains a conservative set of genes in central part and genus- and species-specific genes near its ends, bounded by inverted terminal repeats. To the best of our knowledge, to date, TeAdV-1 has the shortest known ITR (25 bases) within the *Adenoviridae* family closest by length to the *Siadenovirus* representatives (Supplementary Table S2). TeAdV-1 closest relative, duck atadenovirus 1, has an ITR more than 2 times longer (53 nucleotides [60]. Unfortunately, to date, information on the functional significance of the length and sequence of the ITR region is limited and primarily concerns synthetic adenoviral vectors used to deliver target sequences.

The coding part of the TAdV genome contains all genes expected for AdVs as well as specific for the *Atadenovirus* genus. Genes are known to have splicing (IVa2, pTP, and 33 K) were annotated manually using predicted splice sites. These results require confirmation by sequencing of mRNA transcripts produced in infected cells. Since the authors did not have the opportunity to obtain a viral culture, experimental confirmation of the results obtained remains the goal of future studies. We have also predicted 3D structures for a conserved set of structural proteins (penton base, hexon, fiber, LH2, and LH3) as well as for DNA-polymerase. For all TeAdV proteins, compared with corresponding proteins with known crystal structures belonging to other members of the *Adenoviridae* family, we observed a high level of structural similarity.

At the time this article was being prepared for publication, a partial genomic sequence of adenovirus isolated from tern (GenBank accession MW067004.1), obtained by another research group, was uploaded to the NSBI database with the annotated taxonomic position of the unclassified *Adenoviridae* (unpublished). Comparison of the sequences of the DNA polymerase and hexon genes of MW067004.1 showed high percentages of identity (99.38% and 99.56% accordingly) with corresponding TeAdV-1 genes, indicating that MW067004.1 and the described TeAdV-1 virus belong to the same species. Thus, we propose to move MW067004.1 from unclassified *Adenoviridae* to the genus *Atadenovirus*.

All *Atadenovirus* species sequenced to date contain at least five genes derived from other organisms (their hosts, bacteria, fungi, or other viruses), or the origin of which is unknown [68,69,70]. These genes are diverse in their functions and are not required for the realization of the life cycle of the virus, however, apparently, they may contain evolutionary information about the history of the virus-hosts interactions. The genome of TeAdV-1 contains 8 hypothetical proteins, some of which have similarities with those of DAdV-1, and others—with the hypothetical proteins encoded in PsAdV-3 genomes [59]. This fact is fully consistent with the phylogenetic analysis carried out for the core genome, according to which TeAdV-1 descended from the most recent common ancestor (MRCA) of DAdV-1 and PsAdV-3. However, the genome of TeAdV-1 also contains a unique, hypothetical protein (ORF8) that was not found in the genomes of other organisms. The nucleotide sequence of ORF8 has also not been found in the genomes of organisms sequenced to date, including known sequences of the *Sterna hirundo* genome. Thus, the origin of this hypothetical protein remains unclear and requires further research.

Historically, the name of the *Atadenovirus* genus was chosen due to the high proportion of nucleotides A and T in the genomes of the representatives allocated to it [71]. However, only ruminant adenoviruses sequenced to date demonstrate a low proportion of G + C nucleotides, on average about 34%. Reptilian adenoviruses of this genus show a balanced nucleotide composition for *Snake adenoviruses A* (on average about 50%) and bearded dragon adenovirus 1 (56%) [69] with the exception for lizard adenovirus 2, which has 44% G + C bases. For avian viruses of *Atadenovirus* known to date, similar statistics are observed: about 53% G + C nucleotides for passerine and psittacine adenoviruses, but biased for the DAdV-1 (43%). Tern atadenovirus 1 genome has 34% G + C bases, which is the lowest rate among avian adenoviruses within the genus *Atadenovirus* and more consistent with ruminant ones. This fact, combined with phylogenetic information, does not support the assumption of a tendency towards a decrease in the proportion of G + C bases in *Atadenovirus* genomes [69]. Variations in the composition of nucleotides can be a consequence of the adaptation of each viral species to the host organism. However, a recent study reported no correlation between the nucleotide composition of the pathogen and its host genome for eukaryotic viruses, in contrast to bacteriophages, for which such dependence was revealed [72], showing that changes in nucleotide composition for eukaryotic viruses may be caused by more complex adaptation processes.

In order to establish taxonomic relationships of TAdV-1, we also carried out a phylogenetic study. Most of the previously published phylogenies of the family *Adenoviridae* were obtained using the single-locus approach. To construct a phylogenetic tree, the alignment of the sequences of individual genes or proteins encoded by them, in particular, DNA-dependent DNA polymerase [1,73,74,75], hexon [4,76,77,78], penton base, or fiber-2 [59] is traditionally used.

Single gene-based trees, although generally congruent, often show inconsistency in topology and significant differences in the values of node supports. It is customary to explain this by the limited amount of evolutionary information that can be extracted from the single-locus alignment or by different rates of evolution of sequences at different loci [79,80,81,82,83,84]. Cases in which the use of the single-locus approach led to incorrect classification of AdVs have already been discovered. Thus, it has been shown that hexon gene sequencing can lead to a low-resolution view or even mischaracterization of a type of human AdVs (in particular, HAdV-D), since the gene readily undergoes recombination [85]. In the example of the genus *Atadenovirus*, it can be clearly seen that the limitations of the single-locus approach and the difference in the bioinformatic pipelines and tools used by different research groups lead to inconsistencies in the results of phylogenetic analysis. For instance, despite the high statistical support values in most of the cases considered below, the topology of nodes and branches within the *Atadenovirus* clade differs significantly in various published phylogenetic trees. The topologies obtained based on DNA polymerase sequence alignment published in studies [1] (bootstrap values > 90) and [75] (Bayesian Posterior Probability values > 0.7) are not consistent with each other. The topologies obtained on the basis of hexon sequence alignment published in studies [76] (Bayesian Posterior Probability values = 1), [4] (bootstrap values > 80) and [78] (bootstrap values > 80) are generally consistent but conflict with others topologies.

The idea that a larger number of characters improves phylogenetic accuracy and resolution, pioneered by Hillis in 1996 [86], contributed to the development of phylogenomics. The phylogenomic approach can be superior to single-gene analyses with respect to the resolution of internal branches as well as the position of taxa forming long branches in single-gene analyses [87]. Phylogenomic methods are much less limited by stochastic error or sampling error, which could potentially lead to poorly resolved or poorly supported phylogenetic trees, compared to single-loci methods [88]. Indeed, whole-genome sequence analysis has become the gold standard for the classification of adenoviruses as well [85].

Therefore, to build a phylogenetic tree we used the phylogenomic approach that is potentially able to confidently resolve the conflicts between the single-gene analyses of the *Adenoviridae* family, some of which were mentioned above. We used a reliable pipeline recommended for phylogenomic analysis [88], which includes essential stages of preparation and analysis of sequencing data. This allowed us to obtain a reliable phylogeny of the family *Adenoviridae* and the genus *Atadenovirus*, within which we localized the new virus TeAdV-1.

It is often difficult to determine if a strain belongs to a new species or is a variant of an existing species [89]. The previously mentioned divergence values of DNA polymerase, which is one of the most conserved proteins, were selected by the ICTV as the most important criterion for species delimitation [3]. According to this commonly used criterion, TAtV-1 does not belong to any previously described species of atadenoviruses, demonstrating values of the pairwise similarity of the amino acid sequence of DNA polymerase not exceeding 58.3% (Figure 2). However, there are many other approaches to viral species delimitation. Several approaches and relevant tools that have been successfully used to analyze adenovirus genomes are reviewed in [90]. With the onset of the post-genomic era, the most widespread are phylogenetic analyses as well as tools based on the ANI (Average Nucleotide Identity) index [91], that is, an index of similarity between a given pair of genomes. Unfortunately, the ANI-like methods, similar to methods based on single-locus distances, also have their drawbacks and are not ideal methods for classification or reclassification. The most important disadvantage is that there is no universal threshold, suitable for different organisms. Therefore, it needs to be set a priori in each analysis, which often seems to be difficult, and in some cases, even unsolvable problem. To establish an objective threshold, the existing classification must be complete and correct, which means that it should not contain any errors in the delimitation of taxonomic units.

In this study, to classify species, we used coalescent-based methods such as GMYC and PTP that combine population genetic and phylogenetic theory to provide an objective means for delimitation evolutionarily significant units of diversity. GMYC and PTP were originally designed for the analysis of single-locus data, but are often applied to concatenated multilocus data by postulating a shared genealogical history [92,93,94]. The methods generally perform well, being mostly congruent with each other and with the species partitions inferred from independent data [47], but have been shown to be sensitive to the reconstruction method [95]. This is another argument for the importance of using a reliable phylogenetic tree for the analysis. In addition to the above approaches, the ASAP method was used in this study. Compared to GMYC and PTP, ASAP utilizes a phenetic approach where similar sequences are clustered in the same group/species [47]. The difference in the approaches used to obtain species partitions allows more accurate verification of results obtained using different methods. Indeed, some authors propose that various methods should be applied jointly and the results compared [96]. The main advantage of the methods we use is that they propose de novo species partitions and do not require any a priori-defined intraspecific genetic distances. All the methods we used classified the virus TeAdV-1 as a separate novel species. Unfortunately, we were unable to apply the Birky index as an additional instrument for the classification of TeAdV-1, as this requires more than one genome belonging to the same species. This is the main limitation of this approach. However, we have successfully applied the rule to reclassify isolates KJ675568.1 and MN025529.1. Taking into account also the results of the species delimitation obtained using the programs GMYC and PTP, we suggest that the isolates KJ675568.1 and MN025529.1 need reclassification and should be attributed to individual species. The isolate MN025529.1 was first classified in a study [66]. Based on the results of phylogenetic analysis, the authors classified the virus as a new isolate belonging to the previously described species *Psittacine atadenovirus A* [59]. The percentage of amino acid sequence identity was 90.2% for DNA polymerase and 97.2% for hexon. The authors, however, noted that, based on the criterion of the phylogenetic distance of DNA polymerase amino acid sequences, which suggests a species delimitation threshold of 10–15% [97], some of the viruses identified may have had to be classified as a new species [66]. Our research has confirmed this assumption.

The robust classification of new species as well as the reclassification of previously described species according to reliable standards is an important issue. Therefore, we urge the authors not to rely solely on the criterion of the percent identity of the individual gene sequences as well as to use reliable tools and approaches for constructing phylogenetic trees.

It should be noted that according to the current criteria approved by the ICTV, species designation in the *Atadenovirus* genus depends on at least two of the following characteristics: phylogenetic distance (>10–15%, based on distance matrix analysis of the DNA polymerase amino acid sequence), host range, nucleotide composition, cross-neutralization and gene organization at the right end of the genome [ref]. As noted earlier, in addition to a solid pool of phylogenetic evidence, the TeAdV-1 virus does not share a host with any other described *Atadenovirus* species, has a different nucleotide composition from the most related species (e.g., GC-content), and also contains genes unique to its genome.

Pervasive selection, which we found in the core genes of all tested representatives of AdVs, confirms that the genus *Atadenovirus* (or even higher taxa) undergoes rapid gene evolution throughout the evolutionary history under consideration.

Genes and specific sites under pressure from the long-term positive selection can be significant in the arms race. Indeed, the phenomenon of pervasive selection is generally most prevalent in pathogen evolution and any biological system influenced by evolutionary arms race dynamics (or balancing selection), including adaptive immune escape by viruses [98]. This effect is also known as the Red Queen Hypothesis (RQH). The RQH suggests that the co-evolution of interacting species should drive molecular evolution through continual natural selection for adaptation and counter-adaptation [99,100]. The divergence observed at some host-resistance [101,102,103] and parasite-infectivity [104,105,106,107] genes is consistent with this. Development of the functional genetics of interactions and comparative analyses has also revealed that fast-evolving genes are commonly those at the interface of biotic interactions [108]. For instance, in a recent study of the ACE2 receptors, which are proteins that SARS-CoV and SARS-CoV-2, bind to invade the host cell, the gene was found under intense selection pressure in bats and positive selection in other selected mammalian hosts [109]. Binary antagonistic co-evolution is likely to be a major driver of evolutionary change within species.

We found sites under the pressure of pervasive positive selection in the genes encoding the following proteins of *Atadenovirus* representatives: DNA replication machinery (Pol, DBP, pTP) and capside proteins (III (penton base), pIIIa). Such sites are evolutionary hotspots under the constant pressure of adaptive selection and, summarizing all of the above, can be directly involved in antagonistic communication between the virus and host cells (Supplementary Tables S6 and S7). They also can be of epidemiological significance as they can hypothetically be used to predict potential antigenic determinants. It is known that epitope mutations are predominantly under positive selection because they affect the antigenic characteristics of a strain [104,105,110,111,112,113,114]. It should be noted that identifying specific sites is a rather difficult task. It is known that modern methods for site prediction often cannot reliably identify adaptive sites [115]. This may explain the inconsistency of the site detection results by two different methods: Bayesian (CODEML) and maximum likelihood (FEL). To understand adaptive evolution, some form of empirical confirmation is necessary. Nevertheless, the obtained data can be used as preliminary information for planning further experiments.

The episodic positive selection that we found in the TeAdV-1 genome confirms that the virus underwent rapid evolution. We found sites under the pressure of episodic positive selection (*PP* ≥ 0.7) in the genes, encoding the following proteins of the TAtV-1: DNA replication machinery (Pol, pTP), DNA packaging machinery (pIVa2), and 100 K protein. The specific sites we discovered are under the pressure of positive selection (ω > 1) in the TeAdV-1 genome, while in the genomes of other representatives of *Atadenovirus* genus the sites mentioned above are under the pressure of negative selection (ω < 1) or evolve neutrally (ω = 1). Such independent events of adaptive evolution might be associated with the speciation process and adaptation to a new host. It has already been shown that positive selection can be associated with crossing the species barrier. As an example, episodic events of positive selection in the molecular evolution of bats rabies virus were detected during the repeated host shifts [107]. Our assumption that a host shift could be the driver of the TeAdV-1 rapid evolution looks plausible and is indirectly confirmed by the fact that among the most related species of *Atadenovirus* in the phylogenetic tree are species that infect birds (*Psittacine atadenovirus A, Duck atadenovirus A*). It is well known that host shifts tend to occur between related species [116].

## Figures and Tables

**Table 1:** Predicted protein-coding genes and methionine-initiated ORFs of TeAdV-1 and syntenic genes of phylogenetically closely related duck adenovirus 1 (DAdV-1) [4] and psittacine adenovirus 3 (PsAdV-3) [59].

TeAdV-1	Gene		Strand	Size (aa)	DAdV-1	PsAdV-3
p32 K	236	1174	−	312	p32 K	p32 K
LH2	1209	1625	+	138	LH2	E1B protein, small T-antigen
LH1	1656	2804	+	382	E1B 55 K	
IVa2 protein	2875	3696	−	296	IVa2 protein	IVa2 protein
	4614	4682				
DNA polymerase	3945	7181	−	1078	DNA polymerase	DNA polymerase
pTP	7157	8950	−	602	pTP	pTP
	11680	11694				
52 K protein	8985	9971	+	328	52 K protein	52 K protein
pIIIa protein	9955	11664	+	569	pIIIa protein	pIIIa protein
penton base protein	11704	13062	+	452	penton base protein	penton base protein
pVII protein	13104	13559	+	151	pVII	pVII
pX protein	13568	13765	+	65	pX	pX
pVI protein	13800	14435	+	211	pVI	pVI
hexon protein	14456	17188	+	910	hexon protein	hexon protein
protease	17185	17790	+	201	protease	protease
DNA-binding protein	17809	18951	−	380	DNA-binding protein	DNA-binding protein
100 K protein	19005	20924	+	639	100 K protein	100 K protein
22 K protein	20758	20982	+	75		
33 K protein	20758	20973	+	150	33 K protein	33 K protein
	21059	21292				
pVIII protein	21323	22129	+	268	pVIII protein	pVIII protein
U-exon	22142	22306	−	54	U-exon	U-exon
fiber protein	22324	24369	+	681	fiber protein	fiber 2 protein
E4.3 protein	24383	25273	−	296	34 K-2	E4.3 protein
E4.2 protein	25221	26027	−	268	34 K-1	E4.2 protein
E4.1 protein	25948	26385	−	145		E4.1 protein
ORF8	26628	26870	−	80		
ORF7	26888	27451	−	187		
ORF1	27508	27966	+	152		
ORF2	28072	28359	+	95		
ORF3	28366	28698	+	110		
ORF6	28872	29558	−	228		
ORF5	29658	30296	−	212		
ORF4	30424	31146	+	240		

**Table 2:** The list of the core genes of the adenoviruses is determined based on the analysis of groups of orthologous genes of the studied set of genomes of the type representatives of the family.

Gene	Annotation
100 K protein	participation in the transport of hexon monomers to the nucleus and trimerization [61]
23 K protein (endopeptidase, protease)	participation in the cleavage of some AdV precursor proteins [62,63]
52 K protein	participation in the packaging of the viral DNA into the capsid [62,63]
DBP (DNA-binding protein)	participation in the elongation phase of AdV DNA replication by unwinding the template [64]
hexon	major capsid protein [62,63]
III (penton base)	major capsid protein [62,63]
pIIIa	minor capsid protein [62,63,65]
IVa2	participation in the packaging of the viral DNA into the capsid [62,63]
Pol (DNA polymerase)	participation in the elongation phase of AdV DNA replication [64]
pTP (preterminal protein)	the protein primer for AdV DNA replication [64]
pVI	minor capsid protein [62,63,65]
pVIII	minor capsid protein [62,63,65]

**Table 3:** Species delimitation schemes were obtained using the ASAP, PTP and GMYC approach. The following abbreviations are used: *Bovine adenovirus F* (BoAdV-F), *Ovine atadenovirus D* (OvAdV-D), *Deer atadenovirus A* (OdAdV-A), *Bovine atadenovirus D* (BoAdV-D), *Bovine atadenovirus E* (BoAdV-E), *Lizard atadenovirus B* (LiAdV-B), *Lizard atadenovirus A* (LiAdV-A), *Psittacine atadenovirus A* (PsAdV-A), *Duck atadenovirus A* (DAdV-A). The last row contains the total number of partitions obtained when analyzing 26 representatives of the *Atadenovirus* genus.

ASAP	PTP	GMYC (Single-Threshold)	GMYC (Multiple-Threshold)
TAdV-1	TAdV-1	TAdV-1	TAdV-1
LC606503.1 BoAdV-F LC597488.1 BoAdV-F MN901942.2 BoAdV-F	LC606503.1 BoAdV-F LC597488.1 BoAdV-F MN901942.2 BoAdV-F	LC606503.1 BoAdV-F LC597488.1 BoAdV-F MN901942.2 BoAdV-F	LC606503.1 BoAdV-F LC597488.1 BoAdV-F MN901942.2 BoAdV-F
U40839.3 OvAdV-D	U40839.3 OvAdV-D	U40839.3 OvAdV-D	U40839.3 OvAdV-D
MK537328.1 OdAdV-A KY748210.1 OdAdV-A KY468403.1 OdAdV-A KY468402.1 OdAdV-A MK343439.1 OdAdV-A KY468406.1 OdAdV-A KY468407.1 OdAdV-A KY468404.1 OdAdV-A KY468405.1 OdAdV-A	MK537328.1 OdAdV-A KY748210.1 OdAdV-A KY468403.1 OdAdV-A KY468402.1 OdAdV-A MK343439.1 OdAdV-A KY468406.1 OdAdV-A KY468407.1 OdAdV-A KY468404.1 OdAdV-A KY468405.1 OdAdV-A	MK537328.1 OdAdV-A KY748210.1 OdAdV-A KY468403.1 OdAdV-A KY468402.1 OdAdV-A MK343439.1 OdAdV-A KY468406.1 OdAdV-A KY468407.1 OdAdV-A KY468404.1 OdAdV-A KY468405.1 OdAdV-A	MK537328.1 OdAdV-A KY748210.1 OdAdV-A KY468403.1 OdAdV-A KY468402.1 OdAdV-A MK343439.1 OdAdV-A KY468406.1 OdAdV-A KY468407.1 OdAdV-A KY468404.1 OdAdV-A KY468405.1 OdAdV-A
AF036092.3 BoAdV-D JQ345700.1 BoAdV-E	AF036092.3 BoAdV-D	AF036092.3 BoAdV-D JQ345700.1 BoAdV-E	AF036092.3 BoAdV-D
	JQ345700.1 BoAdV-E		JQ345700.1 BoAdV-E
MT050041.1 LiAdV-B	MT050041.1 LiAdV-B	MT050041.1 LiAdV-B	MT050041.1 LiAdV-B
KJ156523.1 LiAdV-A	KJ156523.1 LiAdV-A	KJ156523.1 LiAdV-A	KJ156523.1 LiAdV-A
KJ675568.1 PsAdV-A MN025529.1 PsAdV-A	KJ675568.1 PsAdV-A	KJ675568.1 PsAdV-A MN025529.1 PsAdV-A	KJ675568.1 PsAdV-A
	MN025529.1 PsAdV-A		MN025529.1 PsAdV-A
KJ452170.1 DAdV-A KJ452171.1 DAdV-A	KJ452170.1 DAdV-A KJ452171.1 DAdV-A	KJ452170.1 DAdV-A KJ452171.1 DAdV-A	KJ452170.1 DAdV-A KJ452171.1 DAdV-A
KF286430.1 DAdV-A KJ452172.1 DAdV-A MT646045.1 DAdV-A MN310513.1 DAdV-A	KF286430.1 DAdV-A KJ452172.1 DAdV-A MT646045.1 DAdV-A MN310513.1 DAdV-A	KF286430.1 DAdV-A KJ452172.1 DAdV-A MT646045.1 DAdV-A MN310513.1 DAdV-A	KF286430.1 DAdV-A KJ452172.1 DAdV-A
			MT646045.1 DAdV-A
			MN310513.1 DAdV-A
9	11	8	14

**Table 4:** Identified the most reliable candidate sites for positive selection. *PP* values ≥ 0.95 are in bold.

Protein	Amino Acid Coordinate	*PP* (CODEML)	*PP* (HyPhy)
100 K protein	118	0.886	**0.962**
	230	0.807	**0.951**
	450	0.905	**0.972**
	96	0.87	**0.972**
	13	0.903	**0.955**
	162	0.904	**0.950**
	180	0.936	**0.980**
	192	**0.974**	**0.990**
	35	**0.953**	0.946
pIVa2	123	0.937	**0.983**
	137	0.894	**0.982**
	152	0.946	**0.971**
	94	0.935	**0.964**
DNA polymerase	1044	0.862	**0.951**
	366	0.934	**0.973**
	367	0.927	**0.962**
	773	0.941	**0.961**
pTP	145	0.85	**0.961**
	187	0.946	**0.966**
	297	0.859	**0.975**
	405	0.926	**0.952**
	44	0.946	**0.972**
	445	0.939	**0.966**
	565	0.795	**0.950**
	89	0.93	**0.966**
	96	0.939	**0.988**

## Data Availability

Tern atadenovirus 1 complete genome and annotation available in the GenBank database, accession number OL692338.

## Supplementary Materials

The following are available online at https://www.mdpi.com/article/10.3390/microorganisms10010031/s1, Table S1. List of predicted donor and acceptor splice sites in the TeAdV-1 genome. Data obtained using BDGP Splice Site Prediction by Neural Network [24]. The sites involved in the formation of protein sequences are marked with a (*) sign; Table S2. List of genomic sequences of AdVs used in the phylogenetic analysis; Table S3. List of genomic sequences of adenoviruses used in phylogenetic analysis of *Atadenovirus* genus; Table S4. List of reference sequences of *Atadenovirus* genus; Table S5. The length of the ITR region within the *Adenoviridae* family. Based on complete genome sequences, uploaded to the NCBI database (date of accession: 1 September 2021); Table S6. Homologous sequences of hypothetical proteins corresponding to the open reading frames of the variable region of the TAdV, founded by blast search; Table S7. Candidate sites for pervasive positive selection (*PP* ≥ 0.7), identified by CODEML; Table S8. Candidate sites for pervasive positive selection (*p* ≤ 0.1), identified by FEL; Table S9. Identified candidate sites for episodic positive selection. *PP* values ≥ 0.95 are in bold; Figure S1. Predicted 3D structures of proteins where candidate sites for positive selection were identified (marked with red color).
