# Supplementary material for: Complete Genome Sequence, Molecular Characterization and Phylogenetic Relationships of a Novel Tern Atadenovirus
Source: Microorganisms. 2021 Dec 24;10(1):31. doi: 10.3390/microorganisms10010031 (PMC8781740; doi:10.3390/microorganisms10010031)
Supplement: Supplementary file 1 [file microorganisms-10-00031-s001.zip › microorganisms-1518493-supplementary.pdf]

microorganisms

MDPI

Supplementary Materials:

Table S1. List of predicted donor and acceptor splice sites in the TeAdV-1 genome. Data obtained using BDGP Splice Site Prediction by Neural Network [24]. The sites involved in the formation of protein sequences are marked with a (*) sign.

Type	Strand	Start	End	Score	Sequence
Donor	+	388	402	0.46	caggatcgtaaggca
Donor	+	471	485	0.89	ccagcaggtataatg
Donor	+	913	927	0.54	gttttacgtaaatca
Donor	+	1021	1035	0.96	tggtgttgtaagttc
Donor	+	1059	1073	0.99	acaccaagtaagtta
Donor	+	1188	1202	0.95	ttcttttgtaagtgt
Donor	+	1698	1712	0.59	gtaacaggtgcatta
Donor	+	1794	1808	0.98	gttttaagtaagtat
Donor	+	2160	2174	0.68	acaaaaggttggagt
Donor	+	2367	2381	0.89	aaaagtggtatgtgg
Donor	+	2391	2405	0.98	tctgccggtaattat
Donor	+	2495	2509	0.88	taataaggtatatga
Donor	+	2511	2525	0.99	ttagccggtatgtat
Donor	+	3008	3022	0.57	tcatctggtgaataa
Donor	+	3323	3337	0.53	ttagatggtaaagca
Donor	+	4018	4032	0.96	gggcagagtaagtat
Donor	+	4041	4055	0.51	gtactgggtcagttt
Donor	+	4306	4320	0.96	tcacaatgtatgtct
Donor	+	4410	4424	0.87	tagccaagtaagatc
Donor	+	4785	4799	0.7	ttgctgcgtaagaga
Donor	+	4833	4847	0.65	cttggaggtacaatc
Donor	+	4843	4857	0.95	caatcaggtaggtta
Donor	+	4935	4949	0.95	ttttccagtaagtaa
Donor	+	5023	5037	0.87	ccatacagtgagtta
Donor	+	5366	5380	0.95	tatcaaagtaagaaa
Donor	+	5611	5625	0.99	aaatttggtaagtag
Donor	+	5716	5730	1	ttgcatggtaagttt
Donor	+	6324	6338	0.61	tacaaaagtaggttt
Donor	+	6597	6611	0.91	aaacatggtaaaagc
Donor	+	7788	7802	0.42	ttttgaagtaaatca
Donor	+	7959	7973	0.99	gttattggtaagtta
Donor	+	8055	8069	0.93	ctgaaaggtaaacca
Donor	+	8226	8240	1	gtgtaaggtgagtca
Donor	+	8671	8685	0.77	cgaaaaggtattgat
Donor	+	8875	8889	0.92	cgtttaggttagttg
Donor	+	9234	9248	0.87	gataaaggtagaaag
Donor	+	9380	9394	0.52	gattactgtaagaca
Donor	+	9491	9505	0.78	tcaaatggtaacatt
Donor	+	9750	9764	0.96	aaaacaagtacgtat

Type	Strand	Start	End	Score	Sequence
Donor	+	9938	9952	0.87	agatactgtgagtga
Donor	+	10521	10535	0.98	aaatacggtaaattt
Donor	+	10606	10620	0.98	ataacaagtaagtta
Donor	+	10858	10872	0.76	gttaagggtaataaa
Donor	+	12024	12038	0.76	ttttagggtaaaagt
Donor	+	12493	12507	0.93	ccagaaggtatatca
Donor	+	13199	13213	0.91	agttagagtaagaac
Donor	+	13300	13314	0.77	atataaggtatatta
Donor	+	14483	14497	0.72	atcgctggtaggaat
Donor	+	15020	15034	0.67	ggtaaaggtagaata
Donor	+	15341	15355	0.87	aatgcaggtagtttt
Donor	+	15401	15415	0.88	agaaatagtgagtta
Donor	+	15519	15533	0.62	atgatgggtatgaag
Donor	+	15835	15849	0.5	acctaatgtaattga
Donor	+	16028	16042	0.47	cttcctggtacatat
Donor	+	16091	16105	0.49	actttgggtaatgac
Donor	+	16177	16191	0.98	tgatacagtaagtga
Donor	+	16644	16658	0.4	atcaagggtacagtt
Donor	+	16970	16984	0.54	aatatgggtacatta
Donor	+	17652	17666	0.92	aaaaccagtatgtcc
Donor	+	17961	17975	0.82	acttaaagtgaggaa
Donor	+	19560	19574	0.53	tttataggtaaacca
Donor	+	20319	20333	0.96	aatcatggtaattac
Donor	+	20537	20551	0.47	agtatttgtaagcga
Donor	+	20691	20705	0.76	aaaagaggtagtggt
Donor	+	20721	20735	0.98	gacaccggtgaggaa
Donor*	+	20965	20979	0.92	atacctagtaagtaa
Donor	+	21160	21174	0.51	tctacacgtacaagt
Donor	+	21505	21519	0.58	tgccaatgtaaatag
Donor	+	22914	22928	0.89	caatcaggtagagtt
Donor	+	23230	23244	0.59	gcactaagtaattct
Donor	+	23353	23367	0.87	aactatggtaactca
Donor	+	24022	24036	0.93	acaccgggtaaattc
Donor	+	24385	24399	0.98	atttaaagtaagaca
Donor	+	24616	24630	0.91	atacagggtaatttt
Donor	+	25158	25172	0.49	ttcagaggtagtcct
Donor	+	25297	25311	0.98	tcgagaggtaaatgt
Donor	+	25514	25528	0.85	agcaatggtaaaaat
Donor	+	25886	25900	0.93	gtccatggtatgacc
Donor	+	25990	26004	0.68	ccaaccagtaaataa
Donor	+	26743	26757	0.56	atttcaagtaaatga
Donor	+	27268	27282	0.6	gttgcaggtgcttct
Donor	+	27351	27365	0.49	ttgctaggtagatac
Donor	+	28500	28514	0.84	gcgctacgtatgtcg
Donor	+	28659	28673	0.95	tgctgctgtaagtat

Type	Strand	Start	End	Score	Sequence
Donor	+	29557	29571	0.96	atgtcaagtaagggt
Donor	+	30164	30178	0.63	ccaacaggttacatg
Donor	+	30336	30350	0.91	aattactgtgagtta
Donor	+	30532	30546	0.82	aaatgcggtatggat
Donor	+	31275	31289	0.93	aattaaagtaaatat
Donor	-	31161	31147	0.74	ACACTACGTAAATTT
Donor	-	30803	30789	0.84	AGAAATCGTAAGGTG
Donor	-	30773	30759	1	TCTGTCGGTAAGTAC
Donor	-	30731	30717	0.79	CTCTTAGGTTTGTCC
Donor	-	30195	30181	0.8	TTTAGATGTGAGTAC
Donor	-	30170	30156	0.69	CTGTTGGGTAGGTTT
Donor	-	29232	29218	0.99	AAGTAATGTAAGTGA
Donor	-	28851	28837	0.99	TCATCATGTAAGTAT
Donor	-	28494	28480	0.44	ATTCGCAGTAGGTGC
Donor	-	27577	27563	0.73	CAGTGAAGTGAGCAG
Donor	-	27420	27406	0.87	ATTGCAGGTATAACG
Donor	-	27193	27179	0.75	ATACAAGGTCCGTGT
Donor	-	26975	26961	0.96	AAGTATTGTAAGTGA
Donor	-	26458	26444	0.95	GAATATTGTAAGTAT
Donor	-	26140	26126	0.76	TGACCAAGTGTGTAC
Donor	-	26046	26032	0.74	ATACCAGGTGAAACG
Donor	-	25382	25368	0.78	ATGCACTGTAAGAGA
Donor	-	25346	25332	0.77	GCATTACGTATGTTT
Donor	-	25114	25100	0.58	GCTGAAGGTGAATTC
Donor	-	24661	24647	0.99	ATCAGAGGTATGTTT
Donor	-	24461	24447	0.89	TAAGTATGTAAGTGG
Donor	-	23418	23404	0.69	TGCCAAGGTATTATC
Donor	-	23145	23131	0.96	ACTTAAGGTTAGGGG
Donor	-	22980	22966	0.97	ATTTAACGTGAGTTT
Donor	-	22712	22698	0.44	TTAGAAGGTATTTTA
Donor	-	22586	22572	0.73	TCAAGTGGTAACTGT
Donor	-	22378	22364	0.44	CGAAAGGGTACACAG
Donor	-	22152	22138	0.97	TTTTAATGTAAGTAT
Donor	-	22108	22094	0.97	ATTAGAAGTACGTCT
Donor	-	21759	21745	0.85	GGAAATGGTAGGTTA
Donor	-	21745	21731	0.73	ATCTCTAGTGAGTTT
Donor	-	21381	21367	0.75	GTAGCAGGTTGGTAT
Donor	-	21218	21204	0.87	CGTACAGGTATCGTT
Donor	-	21177	21163	0.44	CTAACTTGTACGTGT
Donor	-	20538	20524	0.95	CTGTCAAGTAAGAAT
Donor	-	20513	20499	0.98	CTGAGGGGTGAGTTT
Donor	-	20251	20237	0.92	TGTAAAGGTACCACA
Donor	-	20236	20222	0.53	TCAGATGGTAACATG
Donor	-	20146	20132	0.98	AAATCAGGTAAATTA
Donor	-	19895	19881	0.55	CAATTGCGTATGTAG

Type	Strand	Start	End	Score	Sequence
Donor	-	19859	19845	0.52	TCTATGTGTAAGGCC
Donor	-	19817	19803	0.42	ACAATTTGTAAGAAT
Donor	-	19534	19520	0.99	ATTGGTGGTAAGCCA
Donor	-	19351	19337	0.96	CCGACAGGTAAATTT
Donor	-	18489	18475	0.74	AAATGGGGTAAACAA
Donor	-	18078	18064	0.96	AAATCATGTGAGTTT
Donor	-	17920	17906	0.87	TGCAGAAGTATGTAT
Donor	-	17755	17741	0.93	TGACAATGTGAGTTT
Donor	-	17623	17609	0.72	TCATAATGTAATTAT
Donor	-	17253	17239	0.74	AAGAAAAGTATGTTG
Donor	-	17166	17152	0.76	GCAAAAGGTAGTCTC
Donor	-	16877	16863	0.81	TAAGAGGGTATGGCC
Donor	-	16528	16514	0.45	GTTAGGTGTAAGCAA
Donor	-	16451	16437	0.63	GAGTTAGGTAAAATG
Donor	-	15724	15710	0.72	AAGAAAAGTACGCTG
Donor	-	15665	15651	0.96	AACCAATGTAAGCTG
Donor	-	15451	15437	0.8	CCTATCTGTGAGTTC
Donor	-	15239	15225	0.92	TAACAAGGTGTGAAT
Donor	-	14889	14875	0.98	ATTTGAGGTAATTGA
Donor	-	14827	14813	0.75	TGCAGAAGTAGGTGC
Donor	-	14810	14796	0.66	ATGGATTGTAAGCAG
Donor	-	14655	14641	0.44	TGAATAGGTACAATC
Donor	-	14646	14632	0.49	ACAATCCGTAGGTGC
Donor	-	14516	14502	0.77	CAGAAAGGTATTCCC
Donor	-	14278	14264	0.92	GCAACAGGTATATCA
Donor	-	14165	14151	0.97	CTCTTGTGTAAGTGG
Donor	-	13695	13681	0.82	ATCAAAGGTATCAAT
Donor	-	13224	13210	0.97	CTCTATAGTAAGTTC
Donor	-	12911	12897	0.94	ACAGAAGGTACATTT
Donor	-	12891	12877	0.97	GATCCAAGTAAGAGT
Donor	-	12624	12610	0.95	TGGAACTGTAAGTAA
Donor	-	12499	12485	0.91	CTTCTGGGTCAGTCA
Donor	-	12443	12429	0.83	AGTGCAGGTATATTA
Donor	-	12064	12050	0.72	TAGAAGGGTCAGTTT
Donor	-	11789	11775	0.46	TGCAGAGGTGGGAAA
Donor*	-	11686	11672	0.92	ACGTCAGGTAATATT
Donor	-	11314	11300	0.94	TAGCAGCGTAAGGAT
Donor	-	11050	11036	0.63	GTTGCATGTATGTAA
Donor	-	10887	10873	0.81	TCTAGGTGTGAGTGA
Donor	-	10604	10590	0.44	TTAGAAGGTAATATA
Donor	-	10403	10389	0.93	CCCAAAGGTACAGTT
Donor	-	9911	9897	0.5	TGCAGTCGTAAGGCT
Donor	-	8576	8562	0.66	ATATACAGTAAATAC
Donor	-	7668	7654	0.82	GAAGATGGTTAGTTA
Donor	-	7083	7069	0.46	TTTAAACGTAGGCCT

Type	Strand	Start	End	Score	Sequence
Donor	-	6936	6922	0.67	AGAAATTGTAAGAGT
Donor	-	6081	6067	0.94	AATGAAGGTACAAGG
Donor	-	6060	6046	0.44	ATTTATGGTTAGAGA
Donor	-	5014	5000	0.96	CAACAAGGTTAGACA
Donor*	-	4619	4605	0.8	TTTACAAGTGAGTGG
Donor	-	4583	4569	0.44	GATAGGGGTAAAAAT
Donor	-	4487	4473	0.84	ACTGTAGGTAAACAT
Donor	-	4460	4446	0.61	AATGGTGGTAAATTA
Donor	-	4289	4275	0.98	CAACCAGGTAAATTA
Donor	-	3922	3908	0.5	TGACGTGGTAAAAAT
Donor	-	3696	3682	0.96	GGCAGCGGTAAATCG
Donor	-	3558	3544	0.99	ATAGAAGGTAACTAC
Donor	-	2785	2771	0.66	ACATCAGGTATCATA
Donor	-	2561	2547	0.41	TGTCCATGTAGGAGG
Donor	-	2553	2539	0.99	TAGGAGGGTACGTAG
Donor	-	2160	2146	0.97	TTGTCCAGTAAGTAT
Donor	-	1724	1710	0.62	AGGCCATGTAGGTAA
Donor	-	1483	1469	0.69	GGATCTGGTAAATTT
Donor	-	1297	1283	0.72	TCAAGAAGTAAATCA
Donor	-	1010	996	0.61	GAAATATGTAAGAAA
Donor	-	855	841	0.41	CAACATGGTCAGGAG
Donor	-	502	488	0.51	ATTAGAGGTATTTTA
Donor	-	238	224	0.74	TAAAAAGGTAGAATA
Acceptor	+	410	450	0.4	tacttgctttccttttccaaggagatatcctaaaccgtcaa
Acceptor	+	457	497	0.51	ctagaattatccatccagcaggtataatgtttaaaatacct
Acceptor	+	805	845	0.62	agttgtattgtctacttgtagttgatggaaactgcactcct
Acceptor	+	1129	1169	0.98	ttattttattcttttatagtgagaagaactatactgttt
Acceptor	+	1684	1724	0.81	ctgcttttccatttgtaacaggtgcattacctacatggcct
Acceptor	+	1756	1796	0.51	ctgtttatacaatttctccagatgaaaatataaactatgtt
Acceptor	+	1997	2037	0.72	tcttgaagatgttcctgatagatctgagcatatgcaggttg
Acceptor	+	2284	2324	0.91	agatatgtttctatgtaataggtggtaattggaatagatct
Acceptor	+	2893	2933	0.74	taatttctttatatattgcagttgtgcatatgttctctttc
Acceptor	+	3169	3209	0.81	acttatttgtgttgattccagttggggactaataacgtgac
Acceptor	+	3387	3427	0.89	catctattataattgctataggtcctttttcagctgcacga
Acceptor	+	3399	3439	0.68	ttgctataggtcctttttcagctgcacgagcaaatatgttg
Acceptor	+	3570	3610	0.73	cccatgtcatttgttcctcagtgctgattgtattagattca
Acceptor	+	3765	3805	0.55	tcattttttgttgtgtactagctataattttgttgtatgtt
Acceptor	+	3789	3829	0.75	taattttgttgtatgttacagcgtgattgtataattcatta
Acceptor	+	3841	3881	0.82	atgacatgacgctttttctagaactgtatctagatttggaa
Acceptor	+	4094	4134	0.79	tcagttgtttctccattacagtgaatggttttgatttctga
Acceptor	+	4466	4506	0.65	ttaatcgatgtttacctacagttaacattctttcatgacca
Acceptor	+	4617	4657	0.47	aaatgctctcgtccacgccagaacgaatgatgcgatctgtg
Acceptor	+	4856	4896	0.44	taaataactgttttgttttagtgaagtctgttctaggtaga
Acceptor	+	4871	4911	0.77	ttttagtgaagtctgttctaggtagagatgaacaaataaca
Acceptor	+	4922	4962	0.95	ctgtaatttctgcttttccagtaagtaacttatttaagtct

Type	Strand	Start	End	Score	Sequence
Acceptor	+	5066	5106	0.99	ttaaattttttcttttcagctttttcttttgctttaatg
Acceptor	+	5725	5765	0.8	aagtttatttcattttcgcagaatagtttgtagccttcaac
Acceptor	+	5801	5841	0.8	catcgatgcagtattttatagattctgatattatgtcgtat
Acceptor	+	5831	5871	0.52	ttatgtcgtatgttttgccagcttctggtttgttttctaga
Acceptor	+	5850	5890	0.96	agcttctggtttgttttctagatattctttttcagaggccc
Acceptor	+	5864	5904	0.93	tttctagatattctttttcagaggcccagtatttgcgtgca
Acceptor	+	6124	6164	0.66	ttggtgttaggtttttcatagcttggattcggaagattgag
Acceptor	+	6351	6391	0.91	caatttgttgtttttatttagttcgataatatcttcatatg
Acceptor	+	6415	6455	0.8	tgtcttgcgaactttttccagactgcgatagcaaattcttt
Acceptor	+	6772	6812	0.82	tagtattcgcgttttctgtagttgcagttgtgtatttttga
Acceptor	+	6978	7018	0.92	tgcattaattttttgcatagttctgttatttgatctatag
Acceptor	+	6998	7038	0.62	gttctgttatttgatctatagatgttcctgtaattctttcg
Acceptor	+	7023	7063	0.82	tcctgtaattctttcgttcagatggtgcaatttaatgtaat
Acceptor	+	7142	7182	0.84	ttgctcggtgttttgttacagtgcttgactccgtctccatg
Acceptor	+	7183	7223	0.64	tttgtattgctctacttctagtggcgtcgaacgtatcaagt
Acceptor	+	7267	7307	0.43	ttctaaatgatatttccacagattctacatcaatatcatta
Acceptor	+	7414	7454	0.59	tatctgaattttcacttatagtaacaaagtcatgcctaatt
Acceptor	+	7516	7556	0.97	cctctccattttcgtttctagctcggagtattacttgtgca
Acceptor	+	7766	7806	0.79	ctgggataattcgtcttctagattttgaagtaaatcaacta
Acceptor	+	7826	7866	0.43	gttatattcttgcctactcagaccgacttcacgtcggtctg
Acceptor	+	7867	7907	0.92	acggtcctggtacttctacaggttctgttggttggtacggc
Acceptor	+	7924	7964	0.7	catcgcctacatctgccgcagggcgtcgtcggcgtgttatt
Acceptor	+	8142	8182	0.94	atttcttttatgttctttaaggttgcttctccatagtctgt
Acceptor	+	8157	8197	0.82	tttaaggttgcttctccatagtctgtgattgctgacatagt
Acceptor	+	8861	8901	0.92	cattatacgcctgtcgtttaggttagttgttatttgcatat
Acceptor	+	8964	9004	0.48	aaaaaatgtgtcttgtaacagatgcatccaattatgaagaa
Acceptor	+	9146	9186	0.67	agaagcagttgatatttttagagatcctgaaccaaaaatgt
Acceptor	+	10215	10255	0.64	tttttctgatcttttaattagagttcacaaatataatagta
Acceptor	+	10701	10741	0.66	tgctttatttaatatttatagagatacactgtctgcttcca
Acceptor	+	11135	11175	0.75	attactcagatttttacatagatatgcaaaattcaaatgat
Acceptor	+	11624	11664	0.75	tacatatgttttcacatctagctccaaaacgaggaaaataa
Acceptor	+	11709	11749	0.51	aacgtacaatgctcctcctagaatttttgcgccaacagaag
Acceptor	+	11795	11835	0.66	caacgaatatttattttatagacaacaaaacaagtgatata
Acceptor	+	12009	12049	0.62	taataatagtaactcttttagggtaaaagtaatgattgata
Acceptor	+	12713	12753	0.91	ttgctactcttccagttgtaggaatgcagttgtttccacta
Acceptor	+	12816	12856	0.58	taatacgttggtgttcaatagatttccaactaatgaaatac
Acceptor	+	12896	12936	0.79	aaaatgtaccttctgttacagatcacggccagcagccgttg
Acceptor	+	13547	13587	0.75	attaaatttgtaattttccagatgagaacaagaacctacag
Acceptor	+	13764	13804	0.98	aaatacttttcatttaacagattttgagcgtgaatatggc
Acceptor	+	14435	14475	0.83	aagatagttattttattccagatggagccacagagagaatt
Acceptor	+	14583	14623	0.43	cttatgtagctccttcatcaggagtaactacggatcgttcg
Acceptor	+	15294	15334	0.62	gtttcagggacaattttataggtattatgtattataactct
Acceptor	+	15417	15457	0.42	gttatcaatttttaattgcagaactcacagataggtataaa
Acceptor	+	16861	16901	0.53	ttggccataccctcttattagtgatgttgcggttaaggatc
Acceptor	+	17084	17124	0.95	ctatttggtgtttttgatcaggttgttgtgaatcagcctac

Type	Strand	Start	End	Score	Sequence
Acceptor	+	17267	17307	0.67	gtagatttcctgggtttttagatagtaataagattaaaact
Acceptor	+	17853	17893	0.84	caacaaccttgcgctttttagcgatgggtatatcgccaaat
Acceptor	+	17934	17974	0.99	catttttacttgctttgcagggtcccacttaaagtgagga
Acceptor	+	18007	18047	0.57	ataccacaggtttcttgctagaatcattgcttgaataacat
Acceptor	+	18220	18260	0.41	ttttgcaatctgcctccctagtatggccctatttgcatagt
Acceptor	+	18417	18457	0.62	gctgctgcatgttatcttcaggacatatcattgaattttcc
Acceptor	+	18503	18543	0.64	atacctcttccatcttttaaggcactcattccagcctcact
Acceptor	+	18745	18785	0.96	cacatgttttgattttttaggacctgactaaagatctttt
Acceptor	+	18906	18946	0.63	tctttttcactttctttgaagtgacgggtttctttgaggtt
Acceptor	+	18967	19007	0.96	tgacacggttttccctcacaggagcctgaaaaatacagatg
Acceptor	+	19546	19586	0.68	tgttaatagaaatttttataggtaaaccacaagaaccaaat
Acceptor	+	19693	19733	0.53	ccactttgctgtcatgtatagacagatttatgaatagaaag
Acceptor	+	19927	19967	0.88	acacaatatatttgtttctagtatttatgtggcagacaatt
Acceptor	+	20381	20421	0.62	taatttatgtgctcctcacagaatgccatgttataatacag
Acceptor*	+	21039	21079	0.65	cgtactactttaaattgtaggaaattttggagttcaatca
Acceptor	+	22231	22271	0.98	ttttatccattttgttttaggtaaaattacattaaaagat
Acceptor	+	22303	22343	0.94	tcattatattttaatttacagatgaaacgagcaagaagaga
Acceptor	+	23033	23073	0.91	ttaaacctttaacctttgtagataacaagttagctttagac
Acceptor	+	24448	24488	0.9	cacttacatacttatctatagatactttgtttaatttcaca
Acceptor	+	24469	24509	0.86	atactttgtttaatttcacagtgtcatatttatcagcacat
Acceptor	+	25174	25214	0.76	gtaattttcaactatcatagattttgaaatgaacccatat
Acceptor	+	25357	25397	0.65	tgggaacattttctcttacagtgcatatcactgttattaca
Acceptor	+	25709	25749	0.78	tatagtatttaatttctctagaacccattgtaaaatacaga
Acceptor	+	25823	25863	0.54	caaatcctgtatatattgcaggaggagaaggataatggctg
Acceptor	+	26055	26095	0.52	aactgcaatgttcatttgtagatgaatccaaagcctgctct
Acceptor	+	26809	26849	0.58	aaatcttccatatcttcatagttcagaccacttccataatc
Acceptor	+	27183	27223	0.54	ggaccttgtatagttttgtagttattttcttccattctctt
Acceptor	+	28308	28348	0.7	attttttgtgcaactcaacagaactatagttgcatacagct
Acceptor	+	28725	28765	0.99	attttttattatatttcttaggttgctgcactacgtcaaga
Acceptor	+	30014	30054	0.85	cggaatatattctttgtatagatttcatttttcaataaat
Acceptor	+	30206	30246	0.66	ctataactttgcttatctcagtatttccgccatagcaataa
Acceptor	-	31216	31176	0.77	ACTATTTTTATTAGTTTTTAGTACTTAATGCAGTAGTTTTA
Acceptor	-	31008	30968	0.91	TATATTGTGTGTTCTCCATAGCGAAGGGCGGAAGCACTTAG
Acceptor	-	30745	30705	0.73	ATATGCGCACAATTCTCTTAGGTTTGTCCATGTACTCTGTA
Acceptor	-	30724	30684	0.97	GTTTGTCCATGTACTCTGTAGGTGTACTGTAGAATCCGACT
Acceptor	-	30605	30565	0.92	AATGTGTTTATTTTGTTGTAGGCGAGGAATTCACCGAAAAT
Acceptor	-	30532	30492	0.61	TTGTGATTGTTTTCGCAATAGGAGTATTTCCAGCGAAACAT
Acceptor	-	30142	30102	0.46	TAAGCACTTTGTGTGTAATAGGTGATGTTAATTCACATGCA
Acceptor	-	29683	29643	0.88	ATAATTCACTTGTATTTTCAGATTAAAAAATTTCACATAAG
Acceptor	-	29177	29137	0.55	TACGAACATTTTTCTAGACAGGATTTTGCAAGTTTTGTTTA
Acceptor	-	29073	29033	0.54	AAATAGACAATTTTCTCATAGACGACCGTGTATAGTGTGTA
Acceptor	-	28786	28746	0.97	CTTGTATGATTTTCTTTTCAGTCTTGACGTAGTGCAGCAAC
Acceptor	-	28504	28464	0.44	AGCGCTCTATATTCGCAGTAGGTGCACATCCACAGTTAATA
Acceptor	-	27963	27923	0.67	TGATATGCTTATTTTGGATAGGGAACGAATTGTTCTGCGTT
Acceptor	-	27677	27637	0.87	TAATTCTTTTCTCTTGTGCAGTAACTCGCAATCAAGTTGGG

Type	Strand	Start	End	Score	Sequence
Acceptor	-	27434	27394	1	TTATTTTTTTTTTTATTGCAGGTATAACGTCATAGACCTTC
Acceptor	-	27087	27047	0.44	TTGCTAAGTGTGCTTGTATAGATCATTTGTTTTTGTGGAAT
Acceptor	-	26908	26868	0.48	AAATGTGTGTTTGACTTTTAGTGCTGTGGTTACTGTCAATG
Acceptor	-	26743	26703	0.83	TACTATTTATTCTGTCTGTAGCTATGTACATATCATGTGTT
Acceptor	-	26495	26455	0.58	ACTCCGTTTTTGTAATTATAGAAGGTTTGCTTCTATTGAAT
Acceptor	-	26406	26366	1	ATTTTTGTTTTTCTCTTACAGATGGCTAATGACGTTCGTTA
Acceptor	-	26266	26226	0.46	TTACGCGGTTGGCCCTTTCAGAATTGTATTCCAATTATCTA
Acceptor	-	26162	26122	0.89	TCATACGGTGGCTATTTATAGATGACCAAGTGTGTACATGT
Acceptor	-	25984	25944	0.91	TCCTCTCTATCTCTCATATAGGGTTGGGTGGCAATAGAATG
Acceptor	-	25666	25626	0.9	CAGCACGAGCTTTTTTGATAGATTCTCTTAAAAGATCAAAA
Acceptor	-	24553	24513	0.98	TGTTTTTATGCTTTTTTGCAGTGTGATTGTAAACAGAAATG
Acceptor	-	23758	23718	0.52	TTTTATATCCCATTTGTATAGAATAAAAAATAGATGATGTA
Acceptor	-	23514	23474	0.43	AGTATCTGGATTGTATCGCAGAGCTAATCCATCATTTGAAG
Acceptor	-	23294	23254	0.88	AAATTATTTTCTTTTACATAGAAGTTATTTGAAGTATTTAG
Acceptor	-	23175	23135	0.48	ATTTCCTATGTTTAATCCTAGTTTATCTTTACTTAAGGTTA
Acceptor	-	23159	23119	0.91	CCTAGTTTATCTTTACTTAAGGTTAGGGGTGACATTGGCGC
Acceptor	-	22602	22562	0.69	CATATTGTTATCTTTTTCAAGTGGTAACTGTACTAAACCAT
Acceptor	-	22567	22527	0.8	AACCATACATTTCATTTGCAGATAGTTGACCTGCGCTATCT
Acceptor	-	21818	21778	0.53	TGGTTCCTGTTCCTTGTAAAGATACCCATTCATTTCCACTT
Acceptor	-	21786	21746	0.75	TTTCCACTTTTGACTTGCCAGTTATAAGGAAATGGTAGGTT
Acceptor	-	21662	21622	0.8	GTTGTTCTCCATTACTTGTAGCTATAAAATCGTGTTGATTC
Acceptor	-	21630	21590	0.8	TGTTGATTCTGTGCGCTGTAGGTTGTTTTCCTCGCAGGAAT
Acceptor	-	21614	21574	0.94	TGTAGGTTGTTTTCCTCGCAGGAATGTATGGCATGCCATGC
Acceptor	-	21395	21355	0.78	CAGCGACCTTTCCCGTAGCAGGTTGGTATGACCAAAGATAA
Acceptor	-	21268	21228	0.91	CAGCTTCTTTGCGTCCTCCAGACACTGCAGTGCCTTCTTTT
Acceptor	-	21232	21192	0.94	CTTTTCGTCCCGCTCGTACAGGTATCGTTTTGTAATACTTG
Acceptor	-	20989	20949	0.89	TAATCTTTTATTACTTACTAGGTATTACAGCTTTTGTCTTC
Acceptor	-	20959	20919	0.85	CTTTTGTCTTCTTTTTTGGAGGGGGAGATATGTCATCATCG
Acceptor	-	20827	20787	0.98	CACTGCGCTCCTTTCCAGTAGCTGGGCTCTGAGAGTCCGCC
Acceptor	-	20708	20668	0.87	TACACCACTACCTCTTTTTAGTAATTCCATTTCCCGCCTTA
Acceptor	-	19436	19396	0.98	AAATTTTTGATTGTTTTTTAGTTCTGAAATTACAGGAGCAT
Acceptor	-	19182	19142	0.97	AATCATTTTTCCTGTCATCAGGTGTAAAAATACTTCTTTCT
Acceptor	-	19160	19120	0.62	TGTAAAAATACTTCTTTCTAGTAGCTTAGATATATATAGCA
Acceptor	-	19106	19066	0.58	TGCATCTTTTGATTTTAGTAGAGAACGTAATATTTTAGTTT
Acceptor	-	19064	19024	0.48	TCTTTCAATATGTTTCATCAGGATAGGTTTTTGCAATTCGG
Acceptor	-	19012	18972	0.91	AAATCCATCTGTATTTTTCAGGCTCCTGTGAGGGAAAACCG
Acceptor	-	17468	17428	0.7	CGTTTAATTATATGTTTATAGCTAAAATTGTAATATTTTTC
Acceptor	-	17347	17307	0.81	GCACACCACCACTTTCCCTAGGCCCAGTATTAACTATTGCA
Acceptor	-	17198	17158	0.63	GAACTTCCACTCATGTTGCAGCACTACCGGCAGCAAAAGGT
Acceptor	-	15970	15930	0.77	ATATCTTCCATTTCCCAACAGTTGCGATCTATATTTTAGTC
Acceptor	-	15653	15613	0.85	CTGAAGTATTATTTTTCTTAGAAGCAGTACTTCCTGATACT
Acceptor	-	15290	15250	0.52	TATAATTTGGTCTATTTCCAGCTGATGAACATTGCAAACCA
Acceptor	-	15026	14986	0.43	CTTTACCACCGCTTTTATCAGAATCGATAAATTGAGGTCCT
Acceptor	-	14954	14914	0.51	TAGGTTCAGTGTCACTTATAGTTTGTAACTTTGCAGCATCA
Acceptor	-	14669	14629	0.47	CATTGTCTTCAATTTGAATAGGTACAATCCGTAGGTGCAGT

Type	Strand	Start	End	Score	Sequence
Acceptor	-	13968	13928	0.68	CAATTTGTTTTGCTGTATTAGTTACAAAATTGCCTATTTTA
Acceptor	-	13766	13726	0.55	TTTATTGCTTCGAATTTTTAGCAGCGATGATTGTTCCAGCA
Acceptor	-	13602	13562	0.84	TGACGTTTCGGTCGCCTGTAGGTTCTTGTTCTCATCTGGAA
Acceptor	-	12543	12503	0.55	CTTATTTTCATTGCCATCTAGTTTTACTACATTGTATGATA
Acceptor	-	12499	12459	0.66	CTTCTGGGTCAGTCATTACAGGTTCTATAGAATCTGGAAAT
Acceptor	-	12187	12147	0.81	CATTTTTTTGTCTTCCAACAGTCAAATATTGATCAACTATA
Acceptor	-	12118	12078	0.73	GTACTGTATAATTCCCTTCAGGAATGCTAAGTGTTACCCAA
Acceptor	-	11805	11765	0.88	AATATTCGTTGTATCTTGCAGAGGTGGGAAAGGATTATACG
Acceptor	-	10903	10863	0.95	TCAATACATTTTCCTGTCTAGGTGTGAGTGATTTATTACCC
Acceptor	-	10780	10740	0.9	CATTTTGTATTTCACTTTCAGTTTCTTCAGGAATTTCATTG
Acceptor	-	10771	10731	0.95	TTTCACTTTCAGTTTCTTCAGGAATTTCATTGGAAGCAGAC
Acceptor	-	10621	10581	0.53	CTAACTTACTTGTTATATTAGAAGGTAATATATCACCATCA
Acceptor	-	10177	10137	0.87	TAACTAACTCTTGTATTATAGAATAAACTTTATCGGCCGTG
Acceptor	-	10099	10059	0.94	CATTTAGTTTGTTAGCTATAGGTTGTTTTTTAAAATTCTCA
Acceptor	-	10057	10017	0.82	TAGCATTTTGACTTCTTCCAGCTAAAATGGATTCAACAATT
Acceptor	-	9963	9923	0.57	CGTCGTCATCTTCACTCACAGTATCTTCTTCGTCAGACTCT
Acceptor	-	9948	9908	0.83	TCACAGTATCTTCTTCGTCAGACTCTGGTCCCTCTAATGCA
Acceptor	-	9764	9724	0.81	ATACGTACTTGTTTTTGCTAGTTTATCCGCTGTTGGATAGT
Acceptor	-	9577	9537	0.9	TTTCCTTTATCATCTCTACAGTTAATTTTAGAAAAAATTCT
Acceptor	-	9192	9152	0.61	CTTCTGACATTTTTGGTTCAGGATCTCTAAAAATATCAACT
Acceptor	-	9046	9006	0.95	TGTGACCCGTCTCCTTCGCAGTTAGACACAGTTGATCGAAT
Acceptor*	-	8971	8931	1	CATTTTTTCTATTTTTTATAGATTCGCATTTATCAGGAGTT
Acceptor	-	8956	8916	0.57	TTATAGATTCGCATTTATCAGGAGTTAACAGGCCAGAGCGC
Acceptor	-	8244	8204	0.64	TATTTGACTCACCTTACACAGATGGTTGGTTAAGAACTTTT
Acceptor	-	8075	8035	0.94	ACGAGTTGGTTTACCTTTCAGACTTAGACAAAGAGACAGAG
Acceptor	-	7995	7955	0.47	GAGCTATTAGACACTTTATAGATAACTTACCAATAACACGC
Acceptor	-	7659	7619	0.78	TAGTTAACTTTTTCATTGTAGAACATATTGCAAGCACTTTA
Acceptor	-	7613	7573	0.41	CTTATATATGAAACTTTATAGACATAGAAATTCAAGAAACA
Acceptor	-	6730	6690	0.91	TTATAAAATTTTTTCCAATAGGCTCATGTGAAAATACTAAA
Acceptor	-	4908	4868	0.67	TATTTGTTCATCTCTACCTAGAACAGACTTCACTAAAACAA
Acceptor*	-	3716	3676	0.41	TTACTGTGTATGGTCCAACAGGCAGCGGTAAATCGCAATTT
Acceptor	-	2718	2678	0.47	AATACACTTTGCTTGCTATAGCAGGATCAAGCCTTATATTA
Acceptor	-	2628	2588	0.47	TACAAAAATTTTTTTGTGCAGCTTTAAAATTTTTAATATGC
Acceptor	-	2571	2531	0.9	GAGTATTCCCTGTCCATGTAGGAGGGTACGTAGAATCATCA
Acceptor	-	1617	1577	0.83	TTTTTAATTTTTTCAATGTAGGAAATAATATTTCAGCAACA
Acceptor	-	1578	1538	0.55	CACGATCACACATTTCTTTAGTGTCCTTCTTAAGATATTCT
Acceptor	-	1565	1525	0.43	TTCTTTAGTGTCCTTCTTAAGATATTCTTTGCATTGCGGAC
Acceptor	-	1479	1439	0.88	CTGGTAAATTTTTTACTATAGTTAAAGCCTTATCTACTACA
Acceptor	-	1458	1418	0.43	TTAAAGCCTTATCTACTACAGAACCAGTACATATAGAACTA
Acceptor	-	1227	1187	0.91	CTTCACTTCCTTCGTCCATAGCAGAACACTTACAAAAGAAA
Acceptor	-	564	524	0.5	CAGCTGTTGGAATTGTTATAGATCTAATATTAGGAAGCCCA

Table S2. List of genomic sequences of AdVs used in phylogenetic analysis.

Genbank Accession	Assembly Accession	Species Taxid	Species
U40839.3	GCA_000842705.1	130327	*Ovine atadenovirus D*
AF252854.1	GCA_000844185.1	114424	*Ovine mastadenovirus A*
AF030154.1	GCA_000844245.1	129950	*Bovine mastadenovirus B*
AF083975.2	GCA_000844285.1	190064	*Fowl aviadenovirus D*
J01917.1	GCA_000845085.1	129951	*Human mastadenovirus C*
U46933.1	GCA_000845105.1	190061	*Fowl aviadenovirus A*
AF036092.3	GCA_000845805.1	130499	*Bovine atadenovirus D*
Y07760.1	GCA_000845925.1	10537	*Canine mastadenovirus A*
Y09598.1	GCA_000845945.1	130328	*Duck atadenovirus A*
AJ854486.1	GCA_000845985.1	130310	*Human mastadenovirus D*
L19443.1	GCA_000846685.1	130309	*Human mastadenovirus F*
HQ605912.1	GCA_000846705.1	298334	*Simian mastadenovirus A*
X73487.1	GCA_000846805.1	129875	*Human mastadenovirus A*
AF289262.1	GCA_000846825.1	130371	*Porcine mastadenovirus C*
AY771780.1	GCA_000847325.1	536079	*Human mastadenovirus G*
AY487947.1	GCA_000859665.1	130308	*Human mastadenovirus E*
DQ086466.1	GCA_000880515.1	108098	*Human mastadenovirus B*
EU835513.1	GCA_000884755.1	696859	*Murine mastadenovirus C*
BK000415.1	GCA_000885275.1	129956	*Murine mastadenovirus A*
GU936707.2	GCA_000888635.1	1146875	*Turkey aviadenovirus B*
HM049560.1	GCA_000889615.1	1146873	*Murine mastadenovirus B*
HQ241819.1	GCA_000890695.1	1962299	*Simian mastadenovirus B*
GU188428.1	GCA_000890915.1	190063	*Fowl aviadenovirus C*
JN252129.1	GCA_000893815.1	1146874	*Bat mastadenovirus B*
GU226970.2	GCA_000894355.2	1146877	*Bat mastadenovirus A*
JF510462.1	GCA_000897155.1	1193420	*Goose aviadenovirus A*
HQ605912.1	GCA_000905275.1	1986020	*Simian mastadenovirus G*
HQ913600.1	GCA_000905855.1	1986022	*Platyrrhini mastadenovirus A*
KC693022.1	GCA_000906395.1	1962300	*Simian mastadenovirus C*
KC493646.1	GCA_000907375.1	190062	*Fowl aviadenovirus B*
KF477312.1	GCA_000912195.1	1962301	*Turkey aviadenovirus C*
FJ025931.1	GCA_000913475.1	1986019	*Simian mastadenovirus F*
KF477313.1	GCA_000913655.1	1962302	*Turkey aviadenovirus D*
KJ563221.1	GCA_000920015.1	1986024	*Sea lion mastadenovirus A*
FN824512.2	GCA_000921315.1	1548417	*Pigeon aviadenovirus A*
KJ469653.1	GCA_000923915.1	1534553	*Duck aviadenovirus B*
KJ156523.1	GCA_000923975.1	2003672	*Lizard atadenovirus A*
KM190146.1	GCA_000928615.1	1986021	*Simian mastadenovirus H*
KJ675568.1	GCA_000929035.1	2003673	*Psittacine atadenovirus A*
KP238322.1	GCA_001271155.1	1986023	*Skunk mastadenovirus A*
KT160425.1	GCA_001271175.1	129955	*Equine mastadenovirus B*
KP329563.1	GCA_001430155.1	1986017	*Simian mastadenovirus D*
KP329564.1	GCA_001430595.1	1986018	*Simian mastadenovirus E*
KT698853.1	GCA_001629825.1	2015370	*Bat mastadenovirus C*
JN418926.1	GCA_001714455.1	129954	*Equine mastadenovirus A*
KT698856.1	GCA_001722965.1	2015372	*Bat mastadenovirus D*
KX121164.1	GCA_001831345.1	2169708	*Pigeon aviadenovirus B*
KX871230.1	GCA_001885425.1	2015376	*Bat mastadenovirus G*
KX505867.1	GCA_001904845.1	2170002	*Simian mastadenovirus I*

Genbank Accession	Assembly Accession	Species Taxid	Species
KX961095.1	GCA_002158575.1	2015375	*Bat mastadenovirus F*
KY306667.1	GCA_002163405.1	2170000	*Deer mastadenovirus B*
KY427939.1	GCA_002219645.1	2170003	*Squirrel mastadenovirus A*
KY748210.1	GCA_002355065.1	2169706	*Deer atadenovirus A*
KT862808.1	GCA_002817995.1	190065	*Fowl aviadenovirus E*
BD269513.1	GCA_002818015.1	129953	*Bovine mastadenovirus A*
AF083132.1	GCA_002818095.1	130369	*Porcine mastadenovirus A*
AF258784.1	GCA_002818135.1	242710	*Tree shrew mastadenovirus A*
KX577802.1	GCA_003032855.1	2169709	*Psittacine aviadenovirus B*
LC385827.1	GCA_006415515.1	740971	*Bat mastadenovirus*
AP018374.1	GCA_006425395.1	2039267	Eidolon helvum adenovirus
MK518392.1	GCA_013088485.1	2734581	*Ovine mastadenovirus C*
MH580295.1	GCA_013088515.1	2734577	*Psittacine aviadenovirus C*
LT841149.1	GCA_900098775.1	2170001	*Dolphin mastadenovirus B*

Table S3. List of genomic sequences of adenoviruses used in phylogenetic analysis of *Atadenovirus* genus.

Genbank Accession	Assembly Accession	Species Taxid	Species Name
KY468407.1	GCA_006434375.1	2169706	*Deer atadenovirus A*
KY468406.1	GCA_006434395.1	2169706	*Deer atadenovirus A*
KY468405.1	GCA_006434415.1	2169706	*Deer atadenovirus A*
KY468404.1	GCA_006434435.1	2169706	*Deer atadenovirus A*
KY468403.1	GCA_006434455.1	2169706	*Deer atadenovirus A*
KY468402.1	GCA_006434475.1	2169706	*Deer atadenovirus A*
KJ452173.1	GCA_006441505.1	130328	*Duck atadenovirus A*
KJ452172.1	GCA_006441535.1	130328	*Duck atadenovirus A*
KJ452171.1	GCA_006441555.1	130328	*Duck atadenovirus A*
KJ452170.1	GCA_006441585.1	130328	*Duck atadenovirus A*
KF286430.1	GCA_006445115.1	130328	*Duck atadenovirus A*
MK343439.1	GCA_007310695.1	2169706	*Deer atadenovirus A*
MN025529.1	GCA_008297635.1	1580497	*Psittacine atadenovirus A*
MK537328.1	GCA_008375655.1	2169706	*Deer atadenovirus A*
MN310513.1	GCA_009793125.1	130328	*Duck atadenovirus A*
MT646045.1	GCA_015221715.1	130328	*Duck atadenovirus A*
LC597488.1	GCA_016759075.1	346178	*Bovine adenovirus F*
LC606503.1	GCA_017161635.1	10511	*Bovine adenovirus F*
MN901942.2	GCA_017498715.2	10511	*Bovine adenovirus F*
MT050041.1	GCA_018591195.1	2729647	*Lizard atadenovirus B*
U40839.3	GCF_000842705.1	114430	*Ovine atadenovirus D*
AF036092.3	GCF_000845805.1	70333	*Bovine atadenovirus D*
Y09598.1	GCF_000845945.1	130329	*Duck atadenovirus A*
BK000404.1	GCF_000886775.1	130329	*Duck atadenovirus A*
JQ345700.1	GCF_000904975.1	111167	*Bovine atadenovirus E*
KJ156523.1	GCF_000923975.1	874272	*Lizard atadenovirus A*
KJ675568.1	GCF_000929035.1	1580497	*Psittacine atadenovirus A*
KY748210.1	GCF_002355065.1	78522	*Deer atadenovirus A*
KY427939.1	GCA_002219645.1	2773314	*Squirrel mastadenovirus A*

Table S4. List of reference sequences of *Atadenovirus* genus.

Species	GenBank accession
BAdV-D	AF036092
BAdV-E	JQ345700
DeAdV-A	MK343439
DAdV-1	AC_000004
LiAdV-2	KJ156523
BDAdV-1	MT050041
OAdV-D	U40839
PaAdV-1	MT674683
PsAdV-3	KJ675568
SnAdV-A	Q106414

Table S5. The length of ITR region within *Adenoviridae* family. Based on complete genome sequences, uploaded to the NCBI database (date of accession: 1 September 2021).

TAX	Organism name	Annotated ITR length min	Annotated ITR length max
Atadenovirus/Bovine adenovirus E	Bovine adenovirus 6	42	534
Atadenovirus/Bovine atadenovirus D	Bovine adenovirus 4	59	59
Atadenovirus/Bovine atadenovirus D	Bovine adenovirus 5	67	67
Atadenovirus/Bovine adenovirus F	Bovine adenovirus 7	36	45
Atadenovirus/Deer atadenovirus A	Odocoileus adenovirus 1	40	40
Atadenovirus/Duck atadenovirus A	Duck adenovirus 1	51	53
Atadenovirus/Lizard atadenovirus A	Lizard adenovirus 2	125	125
Atadenovirus/Lizard atadenovirus B	Bearded dragon adenovirus 1	194	194
Atadenovirus/Ovine atadenovirus D	Ovine adenovirus 7	46	46
Atadenovirus/Psittacine atadenovirus A	Psittacine adenovirus 3	45	45
Atadenovirus/Snake atadenovirus A	Snake adenovirus 1	118	118
Aviadenovirus/Duck aviadenovirus B	Duck adenovirus 2	721	721
Aviadenovirus/Fowl aviadenovirus A	Fowl aviadenovirus 1	54	236
Aviadenovirus/Fowl aviadenovirus B	Fowl aviadenovirus 5	86	86
Aviadenovirus/Fowl aviadenovirus C	Fowl aviadenovirus 10	56	56
Aviadenovirus/Fowl aviadenovirus C	Fowl aviadenovirus 4	56	56
Aviadenovirus/Fowl aviadenovirus D	Fowl aviadenovirus 11	72	72
Aviadenovirus/Fowl aviadenovirus D	Fowl aviadenovirus 2	72	72
Aviadenovirus/Fowl aviadenovirus D	Fowl aviadenovirus 3	72	72
Aviadenovirus/Fowl aviadenovirus D	Fowl aviadenovirus 9	72	72
Aviadenovirus/Fowl aviadenovirus E	Fowl adenovirus 8a	77	77
Aviadenovirus/Fowl aviadenovirus E	Fowl adenovirus 8b	77	77
Aviadenovirus/Fowl aviadenovirus E	Fowl aviadenovirus 6	62	62
Aviadenovirus/Fowl aviadenovirus E	Fowl aviadenovirus 7	77	77
Aviadenovirus/Goose aviadenovirus A	Goose adenovirus 4	39	39
Aviadenovirus/Pigeon aviadenovirus A	Pigeon adenovirus 1	56	56
Aviadenovirus/Pigeon aviadenovirus B	Pigeon adenovirus 2	45	45
Aviadenovirus/Turkey aviadenovirus B	Turkey adenovirus 1	95	95
Aviadenovirus/Turkey aviadenovirus C	Turkey aviadenovirus 4	62	62
Aviadenovirus/Turkey aviadenovirus D	Turkey aviadenovirus 5	118	118
Mastadenovirus/Bat mastadenovirus C	Bat mastadenovirus WIV10	51	51
Mastadenovirus/Bat mastadenovirus C	Bat mastadenovirus WIV11	51	51
Mastadenovirus/Bat mastadenovirus C	Bat mastadenovirus WIV9	35	35

TAX	Organism name	Annotated ITR length min	Annotated ITR length max
Mastadenovirus/Bat mastadenovirus D	Bat mastadenovirus WIV12	73	73
Mastadenovirus/Bat mastadenovirus E	Bat mastadenovirus WIV13	61	61
Mastadenovirus/Bat mastadenovirus F	Bat mastadenovirus WIV17	178	178
Mastadenovirus/Bat mastadenovirus G	Bat mastadenovirus WIV18	177	177
Mastadenovirus/Bovine mastadenovirus A	Bovine adenovirus 1	159	159
Mastadenovirus/Bovine mastadenovirus C	Bovine adenovirus 10	368	370
Mastadenovirus/Canine mastadenovirus A	Canine adenovirus 1	161	199
Mastadenovirus/Canine mastadenovirus A	Canine adenovirus 2	196	198
Mastadenovirus/Dolphin mastadenovirus B	Bottlenose dolphin adenovirus 1	220	220
Mastadenovirus/Equine mastadenovirus A	Equine adenovirus 1	101	320
Mastadenovirus/Equine mastadenovirus B	Equine adenovirus 2	253	253
Mastadenovirus/Human mastadenovirus A	Human adenovirus 12	161	164
Mastadenovirus/Human mastadenovirus A	Human adenovirus 18	167	464
Mastadenovirus/Human mastadenovirus A	Human adenovirus 31	86	150
Mastadenovirus/Human mastadenovirus B	Human adenovirus 11	137	137
Mastadenovirus/Human mastadenovirus B	Human adenovirus 11+34	137	137
Mastadenovirus/Human mastadenovirus B	Human adenovirus 14	133	137
Mastadenovirus/Human mastadenovirus B	Human adenovirus 16	114	114
Mastadenovirus/Human mastadenovirus B	Human adenovirus 21	90	114
Mastadenovirus/Human mastadenovirus B	Human adenovirus 21a	98	108
Mastadenovirus/Human mastadenovirus B	Human adenovirus 34	137	137
Mastadenovirus/Human mastadenovirus B	Human adenovirus 35	137	137
Mastadenovirus/Human mastadenovirus B	Human adenovirus 50	114	114
Mastadenovirus/Human mastadenovirus B	Human adenovirus 55	77	143
Mastadenovirus/Human mastadenovirus B	Human adenovirus 7	108	136
Mastadenovirus/Human mastadenovirus B	Human adenovirus B3	111	195
Mastadenovirus/Human mastadenovirus B	Simian adenovirus 21	121	121
Mastadenovirus/Human mastadenovirus B	Simian adenovirus 29	132	132
Mastadenovirus/Human mastadenovirus B	Simian adenovirus 32	131	131
Mastadenovirus/Human mastadenovirus B	Simian adenovirus 33	131	131
Mastadenovirus/Human mastadenovirus C	Human adenovirus 1	103	103
Mastadenovirus/Human mastadenovirus C	Human adenovirus 2	102	721
Mastadenovirus/Human mastadenovirus C	Human adenovirus 5	103	148
Mastadenovirus/Human mastadenovirus C	Human adenovirus 6	102	103
Mastadenovirus/Human mastadenovirus C	Simian adenovirus 34	119	119
Mastadenovirus/Human mastadenovirus D	Human adenovirus 19	154	154
Mastadenovirus/Human mastadenovirus D	Human adenovirus 36	86	86
Mastadenovirus/Human mastadenovirus D	Human adenovirus D9	159	160
Mastadenovirus/Human mastadenovirus E	Human adenovirus 4p	115	115
Mastadenovirus/Human mastadenovirus E	Human adenovirus E4	115	400
Mastadenovirus/Human mastadenovirus E	Simian adenovirus 23	123	123
Mastadenovirus/Human mastadenovirus E	Simian adenovirus 24	132	132
Mastadenovirus/Human mastadenovirus E	Simian adenovirus 25	119	119
Mastadenovirus/Human mastadenovirus E	Simian adenovirus 25.2	126	126
Mastadenovirus/Human mastadenovirus E	Simian adenovirus 26	131	131
Mastadenovirus/Human mastadenovirus E	Simian adenovirus 30	126	126
Mastadenovirus/Human mastadenovirus E	Simian adenovirus 37	127	127

TAX	Organism name	Annotated ITR length min	Annotated ITR length max
Mastadenovirus/Human mastadenovirus E	Simian adenovirus 38	130	130
Mastadenovirus/Human mastadenovirus E	Simian adenovirus 39	126	126
Mastadenovirus/Human mastadenovirus E	Simian adenovirus E22	120	120
Mastadenovirus/Human mastadenovirus F	Human adenovirus 40	161	163
Mastadenovirus/Human mastadenovirus F	Human adenovirus 41	152	160
Mastadenovirus/Human mastadenovirus G	Human adenovirus 52	60	60
Mastadenovirus/Human mastadenovirus G	Simian adenovirus 1	121	121
Mastadenovirus/Human mastadenovirus G	Simian adenovirus 11	71	71
Mastadenovirus/Murine mastadenovirus A	Murine adenovirus 1	93	93
Mastadenovirus/Murine mastadenovirus B	Murine adenovirus 2	121	121
Mastadenovirus/Murine mastadenovirus C	Murine adenovirus 3	126	126
Mastadenovirus/Ovine mastadenovirus C	Ovine adenovirus 8	93	93
Mastadenovirus/Platyrrhini mastadenovirus A	Titi monkey adenovirus ECC-2011	143	143
Mastadenovirus/Porcine mastadenovirus A	Porcine adenovirus 1	147	147
Mastadenovirus/Porcine mastadenovirus A	Porcine adenovirus 3	144	144
Mastadenovirus/Porcine mastadenovirus B	Porcine adenovirus 4	238	238
Mastadenovirus/Sea lion mastadenovirus A	California sea lion adenovirus 1	109	109
Mastadenovirus/Simian adenovirus B	Cynomolgus adenovirus 1	208	208
Mastadenovirus/Simian adenovirus B	Simian adenovirus 50	215	215
Mastadenovirus/Simian adenovirus B	Simian adenovirus A1139	216	216
Mastadenovirus/Simian adenovirus B	Simian adenovirus A1163	220	220
Mastadenovirus/Simian adenovirus B	Simian adenovirus A1173	178	178
Mastadenovirus/Simian adenovirus B	Simian adenovirus A1258	168	168
Mastadenovirus/Simian adenovirus B	Simian adenovirus A1285	166	166
Mastadenovirus/Simian adenovirus B	Simian adenovirus A1296	194	194
Mastadenovirus/Simian adenovirus B	Simian adenovirus A1312	215	215
Mastadenovirus/Simian adenovirus B	Simian adenovirus A1327	215	215
Mastadenovirus/Simian adenovirus B	Simian adenovirus A1335	216	216
Mastadenovirus/Simian mastadenovirus A	Simian adenovirus 3	173	173
Mastadenovirus/Simian mastadenovirus A	Simian adenovirus 48	173	173
Mastadenovirus/Simian mastadenovirus A	Simian adenovirus 6	150	150
Mastadenovirus/Simian mastadenovirus B	Simian adenovirus 49	215	215
Mastadenovirus/Simian mastadenovirus D	Simian adenovirus 13	124	124
Mastadenovirus/Simian mastadenovirus E	Simian adenovirus 16	181	181
Mastadenovirus/Simian mastadenovirus F	Simian adenovirus 18	180	180
Mastadenovirus/Simian mastadenovirus G	Simian adenovirus 20	178	178
Mastadenovirus/Simian mastadenovirus H	Simian adenovirus DM-2014	159	159
Mastadenovirus/Simian mastadenovirus I	Simian mastadenovirus WIV19	95	95
Mastadenovirus/Skunk mastadenovirus A	Skunk adenovirus PB1	181	181
Mastadenovirus/Tree shrew mastadenovirus A	Tree shrew adenovirus 1	166	166
Siadenovirus/Frog siadenovirus A	Frog adenovirus 1	36	36
Siadenovirus/Skua siadenovirus A	Skua adenovirus 1	30	30
Siadenovirus/Turkey siadenovirus A	Turkey adenovirus 3	40	40

Table S6. Homologous sequences of hypothetical proteins corresponding to the open reading frames of the variable region of the TAdV, founded by blast search.

TeAdV-1 ORF	BLAST Best Hit	% Cover	% Identity
ORF1	hypothetical protein [DAdV-1] QOS14199.1	96%	41.10%
ORF2	hypothetical protein [DAdV-1] NP_044721.1	86%	35.71%
ORF3	hypothetical protein [DAdV-1] AJA72346.1	90%	28.00%
ORF4	hypothetical protein [PsAdV-3] QEJ80748.1	96%	32.03%
ORF5	hypothetical protein [PsAdV-3] QEJ80748.1	95%	30.56%
ORF6	hypothetical protein [PsAdV-3] QEJ80748.1	96%	37.00%
ORF7	hypothetical protein [DAdV-1] QTH80092.1	87%	43.90%
ORF8	-	-	-

Table S7. Candidate sites for pervasive positive selection (PP ≥ 0.7), identified by CODEML.

Protein	Amino Acid Coordinate	PP
Pol	586	0.842
Pol	280	0.761
Pol	83	0.758
Pol	251	0.75
pTP	334	0.755
pTP	230	0.718
III (penton base)	302	0.724

Table S8. Candidate sites for pervasive positive selection ($P \leq 0.1$), identified by FEL.

Protein	Amino Acid Coordinate	P
Pol	177	0.0948
Pol	398	0.0914
Pol	598	0.0352
Pol	624	0.0530
Pol	775	0.0759
Pol	780	0.0090
Pol	781	0.0965
Pol	832	0.0169
pIIIa	301	0.0829
DBP	187	0.0573

Table S9. Identified candidate sites for episodic positive selection. PP values ≥ 0.95 are in bold.

Protein	Amino Acid Coordinate	PP (CODEML)	PP (HyPhy)
100K protein	107	0.818	0.843
100K protein	118	0.886	**0.962**
100K protein	149	0.880	0.937
100K protein	205	0.865	0.895

Protein	Amino Acid Coordinate	PP (CODEML)	PP (HyPhy)
100K protein	222	0.890	0.750
100K protein	230	0.807	**0.951**
100K protein	240	0.843	0.863
100K protein	258	0.763	0.931
100K protein	321	0.867	0.882
100K protein	356	0.770	0.717
100K protein	395	0.876	0.929
100K protein	43	0.873	0.929
100K protein	448	0.800	0.769
100K protein	450	0.905	**0.972**
100K protein	487	0.841	0.833
100K protein	519	0.777	0.790
100K protein	96	0.870	**0.972**
52K protein	121	0.814	0.919
52K protein	126	0.844	0.926
52K protein	13	0.903	**0.955**
52K protein	145	0.786	0.891
52K protein	160	0.778	0.714
52K protein	162	0.904	**0.950**
52K protein	180	0.936	**0.980**
52K protein	192	**0.974**	**0.990**
52K protein	292	0.772	0.815
52K protein	320	0.803	0.851
52K protein	35	**0.953**	0.946
52K protein	99	0.872	0.905
DBP	319	0.820	0.836
pIIIa	142	0.716	0.700
pIIIa	299	0.789	0.947
pIVa2	109	0.882	**0.950**
pIVa2	123	0.937	**0.983**
pIVa2	137	0.894	**0.982**
pIVa2	152	0.946	**0.971**
pIVa2	297	0.858	0.845
pIVa2	55	0.742	0.820
pIVa2	56	0.785	0.861
pIVa2	68	0.894	0.949
pIVa2	94	0.935	**0.964**
Pol	1044	0.862	**0.951**
Pol	1066	0.857	0.874
Pol	131	0.944	0.934
Pol	177	0.793	0.869
Pol	18	0.919	0.863
Pol	212	0.852	0.945
Pol	224	0.821	0.925
Pol	274	0.708	0.718
Pol	341	0.909	0.929
Pol	366	0.934	**0.973**
Pol	367	0.927	**0.962**
Pol	452	0.866	0.885
Pol	514	0.762	0.768

Protein	Amino Acid Coordinate	PP (CODEML)	PP (HyPhy)
Pol	650	0.854	0.932
Pol	687	0.801	0.735
Pol	742	0.796	0.736
Pol	773	0.941	**0.961**
Pol	900	0.860	0.860
pTP	115	0.853	0.923
pTP	145	0.850	**0.961**
pTP	162	0.804	0.842
pTP	178	0.856	0.914
pTP	187	0.946	**0.966**
pTP	208	0.882	0.886
pTP	236	0.793	0.833
pTP	242	0.913	0.820
pTP	297	0.859	**0.975**
pTP	405	0.926	**0.952**
pTP	41	0.901	0.947
pTP	44	0.946	**0.972**
pTP	445	0.939	**0.966**
pTP	447	0.908	0.783
pTP	469	0.750	0.905
pTP	485	0.777	0.829
pTP	50	0.904	0.844
pTP	550	0.892	0.899
pTP	554	0.762	0.948
pTP	561	0.767	0.787
pTP	565	0.795	**0.950**
pTP	569	0.868	0.869
pTP	89	0.930	**0.966**
pTP	96	0.939	**0.988**
pVIII	110	0.828	0.817
pVIII	115	0.802	0,888
pVIII	32	0.859	0.927

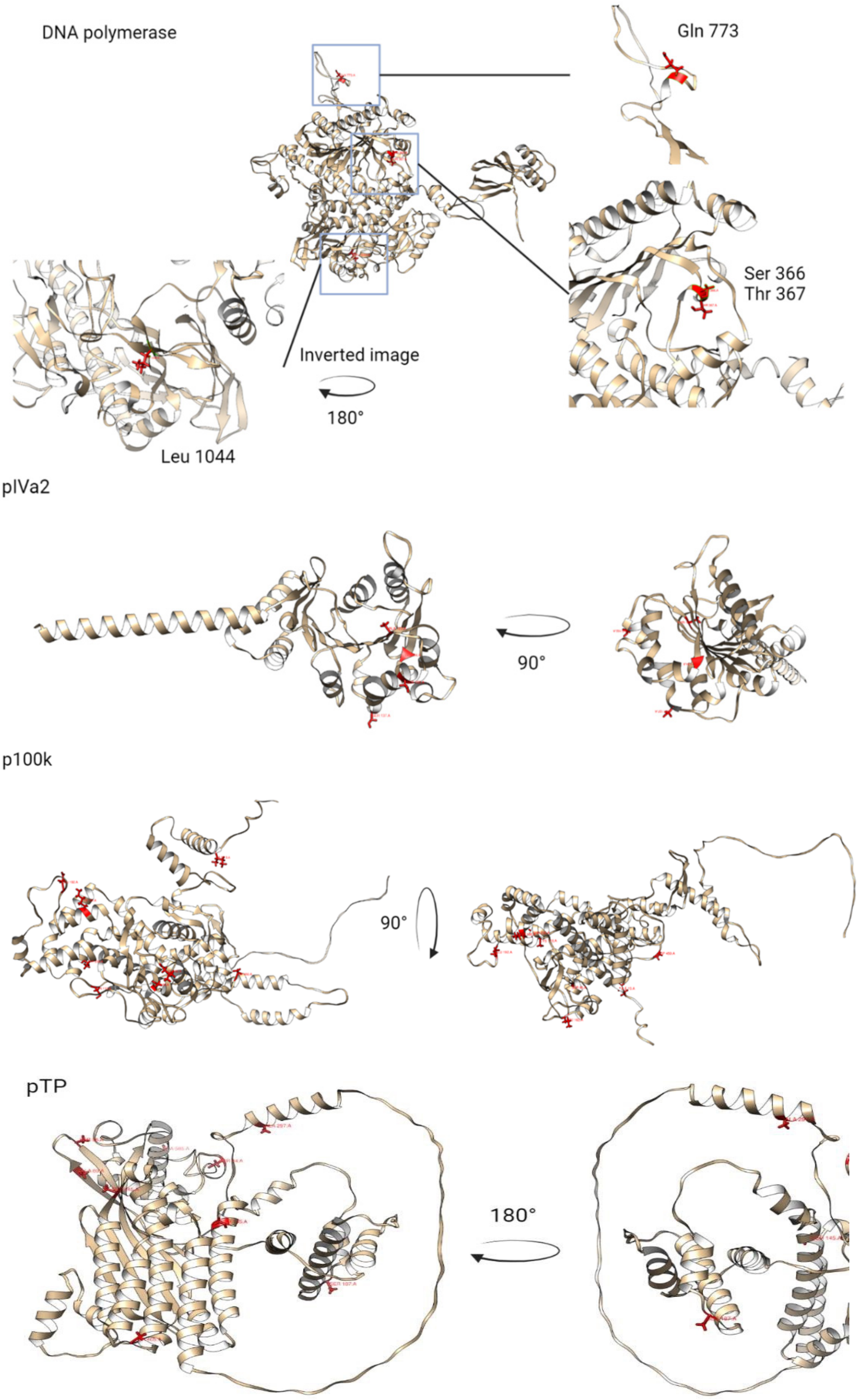

Figure S1. Predicted 3D structures of proteins where candidate sites for positive selection were identified (marked with red color).
